# Estimating and displaying population attributable fractions using the *R* package: graphPAF

**DOI:** 10.1007/s10654-024-01129-1

**Published:** 2024-07-06

**Authors:** John Ferguson, Maurice O’Connell

**Affiliations:** Biostatistics Unit, HRB Clinical Research Facility Galway, University of Galway, Galway City, Ireland

**Keywords:** Population attributable fraction, Impact fraction, Continuous exposure, Directed acyclic graph, Bayesian network

## Abstract

Here we introduce graphPAF, a comprehensive R package designed for estimation, inference and display of population attributable fractions (PAF) and impact fractions. In addition to allowing inference for standard population attributable fractions and impact fractions, graphPAF facilitates display of attributable fractions over multiple risk factors using fan-plots and nomograms, calculations of attributable fractions for continuous exposures, inference for attributable fractions appropriate for specific risk factor $\rightarrow$ mediator $\rightarrow$ outcome pathways (pathway-specific attributable fractions) and Bayesian network-based calculations and inference for joint, sequential and average population attributable fractions in multi-risk factor scenarios. This article can be used as both a guide to the theory of attributable fraction estimation and a tutorial regarding how to use graphPAF in practical examples.

## Introduction

Population attributable fractions (PAFs) measure the extent that the population prevalence or incidence of a particular disease is affected by a known disease risk factor (typical examples of risk factors are smoking, exercise or air pollution). The most straightforward examples of attributable fractions pertain to risk factors that can be eliminated from the population, at least in theory. For instance, one could imagine a population similar to Ireland in almost every way (for instance having similar demographics, culture, a similar health system and so on), except that nobody smoked. How might the rate of heart failure in hypothetical non-smoking Ireland compare to the observed rate of heart failure in Ireland? If the PAF for heart disease attributable to smoking in Ireland is 12% (as was estimated in [1]), 12% of the cases of heart failure that occur in Ireland, would be avoided in an imaginary version of Ireland where nobody smoked. Despite being a hypothetical construct, population attributable fractions are important metrics for determining how pertinent particular risk factors are in determining disease, as well as for ranking differing risk factors as targets for health interventions.

There are a number of currently available *R* packages for estimating attributable fractions under various study designs, mostly designed for the standard setting that considers population-level elimination of a single binary-valued risk factor. paf implements methods described in [2] and concentrates on estimation under cohort designs using a proportional hazards model. attribrisk, [3], estimates attributable fractions in matched and unmatched case–control designs. More recently, AF and stdReg described in [4] and [5] enable estimation of PAF in cross-sectional, case–control and cohort settings. pifpaf, [6] specialises on estimation of PAF using cross-sectional summary data over several independent populations. The new R package graphPAF described here also estimates PAF for cross sectional, case control and cohort study designs under random samples. Unlike some of the aforementioned packages, graphPAF also can estimate PAF for multi-category risk factors and under survey data collection schemes. It also enables PAF calculations in more complicated settings as we describe below.

In the case that many risk factors are under consideration, differing kinds of analyses may be of interest. graphPAF implements fan-plots and nomograms that graphically display the inter-reationships between attributable fractions, relative risk and risk factor prevalence for multiple risk factors, as described in [7]. These analyses can be useful to identify clusters of risk factors that behave similarly, in producing visual rankings of disease burden attributable to differing risk factors, and sometimes in visualizing the effects of interventions.

Joint PAF refers to collective disease burden represented by a group of risk factors (and involves consideration of a hypothetical population where all risk factors in the group were eliminated). Sequential PAFs examine incremental effects on population disease prevalence when each of the risk factors in the group is eliminated in some order. Average PAFs (literally an average of all possible sequential PAFs for each risk factor), allow partitioning a joint PAF into contributions for each risk factor. Previous R implementations of average, sequential and joint PAF (for example the R package averisk, [8]), have been agnostic to the causal structure linking risk factors to disease, which will result in biased estimation in scenarios where multiple risk factors of interest are on the same causal pathway (for instance if smoking affects blood pressure which affects disease, smoking and blood pressure would be considered to be on the same pathway). In contrast, graphPAF can incorporate known risk factor/risk factor and risk factor/disease relationships using a causal Bayesian network model [9], facilitating asymptotically consistent estimation of various PAFs, pertaining to multiple risk factors, in this scenario.

Referring to the putative pathway: ‘smoking $\rightarrow$ blood pressure $\rightarrow$ heart disease’ mentioned above, one might wonder about the extent to which this particular pathway contributes to heart disease. This is measured by the pathway-specific population attributable fraction (PS-PAF), [10], which can be also calculated by graphPAF. Smoking may affect heart disease by mechanisms other than through blood pressure; provided data is available, pathway-specific attributable fractions can also be used to determine the most important pathways through which smoking affects disease. The previous R package causalPAF, [11], can be used to calculate pathway-specific PAFs, however we have updated the estimation routine to be more efficient and robust in graphPAF.

In the case of continuous risk factors or exposures, zero exposure or alternatively elimination of the risk factor can be nonsensical to consider. Consider body mass index (BMI) as an example; zero BMI is obviously unattainable, and extremely low BMI might be as detrimental to one’s health as high BMI. Versions of attributable fractions appropriate in these settings, that allow valid comparisons of disease burden across differing exposures and don’t resort to categorization, are described in [12]. These metrics are also implemented in graphPAF.

In summary, graphPAF extends and consolidates existing packages for PAF estimation in multiple ways. In this manuscript, we describe its features in more depth, interweaving between the theory for PAF estimation and using graphPAF in practice.

The manuscript proceeds as follows. Section “Basic PAF estimation” deals with standard PAF problems, considering a single categorical risk factor, that is either binary valued, or has multiple risk-elevating categories and a reference level. We begin Section “Basic PAF estimation” with definitions of PAF, using the potential outcomes framework, for prevalent and incident disease. We briefly describe the causal exchangeability conditions that are necessary to achieve statistically consistent estimators, and the associated estimators that are applicable under cross-sectional, case–control cohort, survey designs and summarised data. Examples of how to implement these estimators with graphPAF are then illustrated. The section concludes with examples of how to use graphPAF to estimate PAFs with survey data, to estimate impact fractions, and to produce attributable fraction fan plots and nomograms. Section “Estimation with continuous exposures” illustrates definitions and estimators for PAF in continuous expopsure settings, and how to implement these using graphPAF. Graphical approaches to visualise and compare versions of PAF across multiple continuous exposures are demonstrated. Section “Pathway-specific PAF calculations” details the idea of a pathway-specific PAF, a measure of disease burden that is attributable to a single causal pathway linking a risk factor, mediator and disease. Again, we briefly describe causal exchangeability conditions necessary for consistent estimation, and illustrate estimation using using graphPAF. In Section “Joint PAF”, we describe issues with calculating joint PAF across a set of risk factors with known causal structure. We illustrate how to consistently estimate joint PAF with graphPAF via a recursive simulation approach when causal structure is known. Section “Sequential and average PAF” extends the discussion in Section “Joint PAF” to the concepts of sequential PAF and average PAF, which are effectively derived by manipulation of joint PAF for differing subsets of risk factors. Since, exact estimators of average PAF maybe infeasible in problems with a large number of risk factors, we describe strategies to derive approximate estimators that utilise random permutations of the risk factors, that are implemented in graphPAF. We conclude the Section and the paper with further discussion regarding computational considerations when calculating sequential and average PAF.

## Basic PAF estimation

In this section, we consider a scenario where the risk factor is either binary, or perhaps multi-category with some level indicating ‘elimination’ for the risk factor. We will define appropriate PAF estimands for cross-sectional, case–control and cohort study designs, and describe causal exchangeability conditions that are needed for estimation.

### Definitions of PAF under differing study designs

#### PAF for prevalent disease

Cross sectional and case control designs can be used to estimate the proportion of prevalent disease that is attributable to a risk factor. Let *Y* denote a binary disease outcome (1 indicating disease) for a randomly selected individual from the population, and $Y_0$ the same binary disease outcome but where the individual is sampled from a hypothetical population with the risk factor eliminated. The population attributable fraction for prevalent disease can be defined as:

1 $$\begin{aligned} PAF = \frac{P(Y=1)-P(Y_{0} = 1)}{P(Y=1)}, \end{aligned}$$

where $P(Y = 1)$ represents the prevalence of disease in the current population, and $P(Y_{0} = 1)$ the prevalence of disease in the hypothetical population with the risk factor eliminated.

#### PAF for incident disease

In longitudinal cohort studies, a cohort of healthy individuals are followed over time with some eventually developing disease. In this setting, a differing kind of population attributable fraction can be estimated where the cumulative incidence of disease for that cohort as a function of time is compared to what the incidence would be if the factor had been eliminated from the cohort:

2 $$\begin{aligned} PAF(t) = \frac{P(T \le t) - P(T_{0} \le t)}{P(T \le t)} \end{aligned}$$

Here random sampling is interpreted as random sampling from the cohort of interest and *PAF*, *Y* and $Y_0$ from (1) are replaced by *PAF*(*t*), $I\{T \le t\}$ and $I\{T_0 \le t\}$, with the random variables *T* and $T_0$ representing time to disease in the current population and under hypothetical elimination of the risk factor. In the setting of competing events, such as death, we can interpret *T* as the time an individual would have developed disease had the competing event not occurred, and the PAF in terms of prevented disease under elimination of the risk factor provided the competing event did not happen. It should be noted that, in the presence of competing events, (2) can only be estimated under limited conditions, such as non-informative censoring of the true survival time, *T*, by the competing event. However we can also incorporate competing events directly in the definition of PAF. Suppose $\Delta$ represents an indicator for the event that happens first with $\Delta =1$ indicating that disease occurred before any other event. We can then write:

3 $$\begin{aligned}{} & {} PAF^*(t) \nonumber \\ {}{} & {} = \frac{P(T \le t \text { and } \Delta =1) - P(T_{0} \le t \text { and } \Delta _{0}=1)}{P(T \le t \text { and } \Delta =1)}\nonumber \\ \end{aligned}$$

Note that as $t \rightarrow \infty$, $PAF^*(t)$ converges to the PAF for disease incidence described in [13]. While (3) may at first seem a more sensible estimand than (2) in the presence of competing events, care must be taken in its interpretation. For instance, if the risk factor leads to early mortality due to other mechanisms than the disease of interest (3) may be negative for large *t* even when the risk factor causes disease. In other words, while (3) is the proportional difference in cumulative incidence in disease by time *t* due to removing the risk factor, it can’t be interpreted as disease-incidence prevented by eliminating the risk factor. In contrast (2) does have an interpretation in terms of prevented hypothetical incidence in the absence of competing events.

#### Conditions for estimation

Note that (1), (2) and (3) are causal entities, and unbiased estimation with observational data requires relatively strong assumptions. For instance, if the random variable $A\in \{0,1,\ldots ,n_A\}$ represents the observed risk factor ($A=0$ coding for elimination), one cannot say that $P(Y=1 \mid A=0)=P(Y_0=1)$ unless the risk factor *A* is randomly assigned. Informal sufficient conditions for the possibility of asymptotically unbiased estimation of (1) are:

1. Unambiguous definition and measurement of the potential outcome: $Y_{0}$, representing risk factor elimination. (This is essentially the famous Stable Unit treated value assumption (SUTVA), first described in [14])
2. The measurement of a collection of covariates *C*, so that for observed value of *C*, $P(Y=1 \mid A=0, C)=P(Y_{0}=1 \mid C)$ (This will be true if within joint strata of the covariates *C*, the risk factor *A* behaves as if it were randomly assigned). The collection *C* is sometimes refered to as a sufficient adjustment set of covariates.
3. $P(A=0 \mid C)>0$ for all possible values for the sufficient adjustment set of covariates, *C*. (Informally, absence of the risk factor is possible, regardless of an individual’s covariate values).
4. The proposed model for disease, conditional on risk factor and covariates, $P(Y=1 \mid A=0, C)$ is correctly specified.

Similar conditions need to be assumed to estimate (2) and (3). The variables *C* are often, but not always, a set of confounders of the risk factor/outcome relationship (that is they are joint causes of *A* and *Y*). We will assume the veracity of these conditions (including the measurement of a sufficient adjustment set *C*) in what follows, although their validity should be carefully considered in any practical application.

### Estimators of PAF

Differing estimators of PAF are appropriate dependent on the study design. In the following, we detail estimation formulae for PAF separately for cross-sectional, case–control, cohort and survey designs. In defining these estimators, the following notation will be used:

$$\begin{aligned} \begin{array}{ll} i \in \{ 1 \dots N \} &{} \text {represent the}\, N \\ &{}\text {sampled individuals}\\ Y_i \in \{0, 1\} &{} \text {represents the disease} \\ &{}\text {outcome for individual}\,i \\ A_i &{} \text {represents the value of the} \\ &{}\text {risk factor in individual}\,i \\ C_i &{} \text {indicates a `sufficient'}\\ &{}\text {vector of covariates in individual}\,i\\ {\hat{P}}(Y=1 \mid A_i,C_i) &{} \text {An estimator for the} \\ &{}\text {probability of disease given} \\ &{} \text {risk factor value,}\,A_i, \text {and }\\ &{}\text {covariate vector,}\,C_i \\ \pi &{} \text {disease prevalence} \end{array} \end{aligned}$$

#### Cross sectional designs

In cross-sectional designs, a random sample $i \in 1 \dots N$, from the population are collected, at a fixed point in time. In this case, the PAF in the population at the time of data collection, (1), can be estimated by:

4 $$\begin{aligned} \frac{\sum _{i = 1}^{N}(\widehat{P(Y=1 \mid A_i,C_i)}-\widehat{P(Y=1 \mid A_i=0,C_i)})}{\sum _{i = 1}^{N}\widehat{P(Y=1 \mid A_i,C_i)}} \end{aligned}$$

(4) is implemented by graphPAF and utilises the parametric g-formula, sometimes referred to as ‘standardisation’, [15], to estimate PAF. Such an approach only requires the specification of a model for the response. Double robust approaches have also been suggested in the literature that require models for both the risk factor and outcome [16].

#### Case–control designs

In case control studies, disease cases are preferentially selected in the sample, and paired with healthy controls, often in a 1–1 fashion. This implies that the sampled individuals will not be representative of the source population, and equation (4) can’t be used directly. However, if disease prevalence, $\pi$, is known, a similar estimator that re-weights contributions for cases and controls using the same weights, $w_i$, with $w_i=1$ in controls, to ensure that the empirical weighted prevalence of disease $\frac{\sum {w_i Y_i}}{\sum {w_i}}=\pi$, is available:

5 $$\begin{aligned} \frac{\sum _{i = 1}^{N}w_{i}(\widehat{P(Y_i=1 \mid A_i,C_i)}-\widehat{P(Y_i=1 \mid A_i=0,C_i)})}{\sum _{i = 1}^{N}w_{i}\widehat{P(Y_i=1 \mid A_i,C_i)}} \nonumber \\ \end{aligned}$$

One can think of this approach as weighted standardisation. (5) could be considered a direct approach to estimation, in that it estimates the counterfactual probability in (1) by averaging estimated observed data probabilities. In addition to re-weighting the contributions for each individual in equation (5), the estimates ${\hat{P}}(Y_i=1 \mid A_i,C_i)$ may need to be adjusted so they reflect, on average, the assumed disease prevalence in the population. This could be achieved by using estimates from a model fit using weighted maximum likelihood, although this is not what graphPAF does. The correction employed by graphPAF is described below, in the subsection Estimation of prevalent and incident PAF”.

If $\pi$ is unknown, (5) can’t be used for estimation in case control studies. Instead, the formula by [17] should be used:

6 $$\begin{aligned}{} & {} 1 - \frac{1}{N_c}\sum _{i \le N:Y_i=1}\frac{\widehat{P(Y=1 \mid A_i=0,C_i)}}{\widehat{P(Y=1 \mid A_i,C_i)}}\nonumber \\ {}{} & {} =1 - \frac{1}{N_c}\sum _{i \le N:Y_i=1}\hat{RR_i}^{-1} \end{aligned}$$

where ${\hat{RR}}_i=P(Y=1 \mid A_i,C_i)/P(Y=1 \mid A_i=0,C_i)$ is the estimated relative increase in disease risk encountered by individual *i* based on their risk factor value $A_i$ and $N_c=\sum _{i\le N}I\{Y_i=1\}$ is the number of cases in the data set. ${\hat{RR}}_i$ can be approximated by the corresponding odds ratio in case–control study designs provided the disease is relatively rare.

#### Surveys

Equation (5) can also be used to estimate prevalent PAF, (1), for datasets collected with surveys, where $w_i$ is proportional to the inverse of the sampling fraction, that is probability that an individual with covariates, $C_i$ is selected from the population into the sample. In this case, the probabilities ${\hat{P}}(Y=1 \mid A_i,C_i)$ should be estimated using weighted maximum likelihood, using weights $w_i$, so they reflect population quantities. See [18] for more details regarding estimating PAF estimation with survey data.

#### Cohort designs

In cohort designs, cox proportional hazard models are often used to estimate (2). Under the proportional hazards assumption, suppose ${\hat{r}}(C_i,A_i)$ is the estimated hazard ratio for an individual with covariates $C_i$ and risk factor $A_i$ compared to their hazard assuming all covariates and risk factors were at reference levels (defined as 0 for continuous covariates). Let $\hat{H_0}(t)$ be an estimate of the cumulative baseline hazard function. graphPAF uses the Kalbfleich-Prentice estimate for the baseline cumulative hazard function, the default in the survival package) for ${\hat{H}}(t)$. *PAF*(*t*) is estimated as:

7 $$\begin{aligned} \hat{PAF(t)} = \frac{\sum _{i = 1}^{N}e^{-\hat{H_0}(t){\hat{r}}(C_i,A_i=0)}-e^{-\hat{H_0}(t){\hat{r}}(C_i,A_i)}}{\sum _{i = 1}^{N}(1-e^{-\hat{H_0}(t){\hat{r}}(C_i,A_i)})} \end{aligned}$$

To estimate $PAF^*(t)$, ${\hat{r}}(C_i,A_i)$ can be replaced in (7) by the estimated Fine Gray subdistribution hazard ratio: ${\hat{r}}^{FG}(C_i,A_i)$ for disease incidence, and $e^{-\hat{H_0}(t)}$ by $e^{-\int _{0}^{t}{\hat{h}}_0^{FG}(u)du}$, where $h_0^{FG}(u)$ is the baseline subdistribution hazard function at time *u*. As described in the estimation section below, these functions can be estimated by prior weighting of the Cox Model.

#### Estimates from summarised data

In the case of a binary risk factor, *A*, Miettinen, [19], showed that equation (1) can be re-expressed as:

8 $$\begin{aligned} PAF = P(A=1 \mid Y=1) \frac{RR_e - 1}{RR_e} \end{aligned}$$

where *Y* and *A* are the indicators for disease and risk factor exposure for a randomly selected individual from the population, and $RR_e = \frac{P(Y_1 = 1\mid A=1)}{P(Y_0 = 1\mid A=1)}$ is the causal relative risk in risk factor exposed individuals. The above formula can be re-expressed as:

9 $$\begin{aligned} PAF = \frac{P(A=1) RR_u}{1 + P(A=1)RR_u} \frac{RR_e - 1}{RR_e} \end{aligned}$$

where $RR_u=P(Y=1 \mid A=1)/P(Y=1 \mid A=0)$ is the unadjusted relative risk, see [20] for details. Under a no-effect modification assumption: that is assuming $\frac{P(Y_1 = 1\mid C=c)}{P(Y_0 = 1\mid C=c)}$ is constant across confounder strata $C=c$, $RR_e$ can be replaced by the conditional relative risk $RR_c = P(Y_1=1 \mid C=c)/P(Y_0=1 \mid C=c)= P(Y=1 \mid A=1,C=c)/P(Y=1 \mid A=0,C=c)$, within any stratum $C=c$. These observations facilitate estimation of prevalent PAF, by replacing $P(A=1)$, $RR_u$ and $RR_e$ in (9) with estimates of risk factor prevalence, and unadjusted and adjusted relative risk from the published literature. Conditions for the approach to be valid, in addition to the three conditions listed under the subsection “Conditions for estimation”, include no-effect modification, so that $RR_e=RR_c$, that ${\hat{RR}}_c$ is been correctly adjusted for confounding, and transportability of relative risks if the estimates of relative risk and PAF pertain to differing populations. Extensions to (9) for risk factors with multiple levels and continuous exposures are given in [20].

Often a simpler approach, based on Levin’s formula, is used to derive estimates of PAF from summary data:

10 $$\begin{aligned} PAF_L = \frac{P(A=1) (RR_c-1)}{1 + P(A=1)(RR_c-1)} \end{aligned}$$

However, $PAF_L$ will only equal equation (1) in special circumstances, such as complete absence of confounding. In general, Levin’s approach will generate asymptotically biased estimators for PAF, even if consistent estimators for $P(A=1)$ and $RR_c$ are plugged into (10).

### General features of the graphPAF package

The main functions used by graphPAF for estimating differing types of population attributable fractions are detailed in Table 1. To estimate PAF with individual data, the user needs to specify a fitted statistical model, usually supplied to the functions in Table 1 through the model argument. As listed in Table 1, the supported R model functions vary depending on the type of PAF, but include glm model objects, that are fit using the binomial family using the log or logit link, lm objects, polr model objects, fit using the MASS package, and clogit or coxph model objects, fit using the survival package. Common required arguments for many of the functions include the dataset used to fit the model, data, the riskfactor of interest, riskfactor, and the reference value for that risk factor, refval that codes for ‘non-exposure’. Other exported functions that are listed in Table 1, include auxilliary functions to assist simulataneous fitting of multiple models, functions for plotting and nomograms, and functions that estimate PAF from summary data.

**Table 1:** The main estimation functions in the graphPAF package and supported model types

Type of PAF	Function	purpose	Supported modeling functions
discrete risk factor	PAF_calc_discrete()	estimate PAF	glm, clogit, coxph
	impact_fraction()	estimate impact fraction	glm, clogit, coxph
	paf_levin()	estimate PAF with summary data	NA
	paf_miettinen()	estimate PAF with summary data	NA
	rf_summary()	create an rf_summary object for plotting nomograms	NA
continuous exposure	plot_continuous()	plot of estimated relationship between exposure and outcome	glm, clogit, coxph
	PAF_calc_continuous()	estimate PAF_q for a continuous exposure	glm, clogit, coxph
mediating pathway	ps_paf()	estimate pathway specific PAF	A list of lm,polr and glm objects for mediators, a glm object for the response
multiple risk factors	data_clean()	Remove redundant variables from dataset, and calculate prevalence-based weights for modeling	NA
	automatic_fit()	Automatic fitting of statistical models for a specified DAG and dataset	NA
	joint_paf()	estimate joint PAF	A list of lm, polr and glm models
	seq_paf()	estimate sequential PAF	A list of lm, polr and glm models
	average_paf()	estimate average PAF	A list of lm, polr and glm models

#### Confidence intervals

As a default, point estimates of PAF are printed to the screen. For individual data, bootstrap-calculated confidence intervals for *PAF* can be requested via ci=TRUE. The Bootstrap is assisted by the R-package boot, with confidence interval calculations being produced by boot::boot.ci. Parallelization over multiple CPU cores is available by setting the option boot.ncpus to an integer above 1. The number of Bootstrap replications can be changed using the argument boot_rep, which has a default of 50. As a default, bias-corrected symmetric confidence intervals are produced, which combine bootstrap estimates of bias and standard error with the point estimate. This default was set with computational speed in mind, and might be increased for smaller datasets where bootstrap sampling is relative easy. Efron and Tibshirani [21] show that 200 bootstrap replications is usually sufficient for bootstrap estimates of standard errors, and consequently for symmetric confidence intervals for normally distributed estimators, but values as low as 25 replications can be useful to assess variability when sampling is expensive. The default type of bootstrap confidence interval, ci_type="norm", can be changed by setting the ci_type argument to "basic", "perc" or "bca". Note that boot_rep should ideally be increased be about 2,000 if this default is changed. In particular, the number of bootstrap replications needs to be set to larger than the number of rows in the dataset if ci_type="bca", otherwise the function will return an error. See the documentation of the "boot" package for more details. When confidence intervals are produced with individual data, using any of the functions PAF_calc_discrete, impact_fraction, PAF_calc_continuous, ps_paf, joint_paf, seq_paf and average_paf, produces additional output in addition to estimates and confidence intervals regarding estimation and inference settings. This extra output can be suppressed by setting the argument verbose=FALSE.

#### Data sets available

Two simulated datasets are provided with the graphPAF package. stroke_reduced is a matched case–control dataset including 10 stroke risk factors for 6,856 stroke cases and 6,856 stroke controls. The simulations were calibrated accorded to probability distributions estimated using a Bayesian network model fitted to real data from the INTERSTROKE project, [22]. Stroke cases and healthy controls, cases being indicated by case=1, are matched by age-group, gender and region. Matched cases and controls share the same value of the strata variables. Risk factors have differing datatypes, including binary (smoking, stress, diabetes, high_blood_pressure, early_stage _heart_disease, exercise), ordinal (alcohol), and continuous (waist_hip_ratio, lipids diet). To illustrate survival analyses, a time to event variable, time>0 and event indicator, event, with 0 indicating right censored observations, are also included. Hordaland_data is a 2nd example of a case control dataset, pertaining to 5,000 individuals with chronic cough and 5,000 controls, with two binary risk factors (urban.rural, occupational. exposure), and one ordinal risk factor smoking .exposure, simulated based on the relationships in [23].

### Estimation of prevalent and incident PAF

The function PAF_calc_discrete estimates PAF for categorical risk factors (binary risk factors, or multiple-category risk factors) collected via cross-sectional, case control and longitudinal cohort designs. As an example, consider estimating the PAF for the variable exercise, a binary indicator for physical inactivity, using the dataframe: stroke_reduced.

First, to deal with the case–control matching, we fit a conditional logistic model to describe the relationships between the prevalence of stroke, exercise and assumed confounders, with the following commands: 
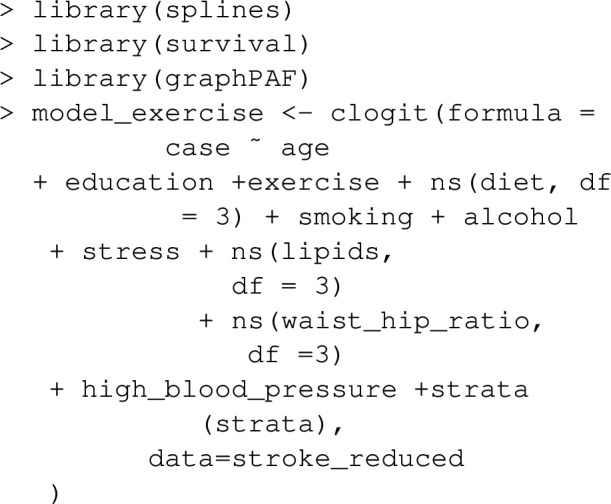
 The PAF for physical inactivity can then be calculated using the function PAF_calc_discrete. Note that the reference value of the risk factor variable in R, that is the value corresponding to no risk factor exposure, is specified below as refval. 
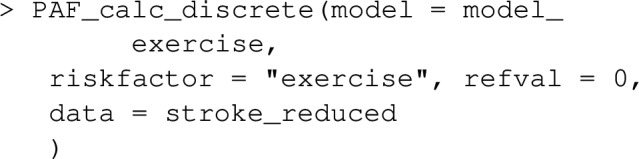

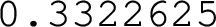
 For case–control datasets such as stroke_reduced, the ‘Bruzzi’ method is recommended as it doesn’t require specification of disease prevalence, provided the disease is relatively rare (the approximation of risk-ratios with odds-ratios might be unacceptably inaccurate otherwise). This is the default method employed by graphPAF. If the prevalence or alternatively the average incidence over a period of time is known, the ‘direct’ approach, as described by equation (5), can also be used to estimate PAF in case–control studies, by specifying calculation_method="D" and a value for prev as arguments, as indicated below. Note that when disease prevalence is specified as $\pi$, graphPAF adjusts predicted probabilities via adding a constant to the linear predictor of the estimated model to ensure that $\sum _{i=1}^{N}w_i{\hat{P}}(Y_i=1 \mid A_i,C_i) / = \sum _{i=1}^{N}w_i \pi$. 
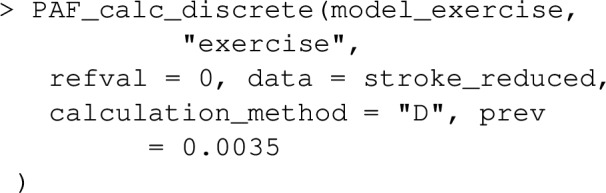

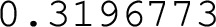
 The above calculation assumes that the yearly incidence (averaged over the cohort) of first stroke is 0.0035, which effectively estimates *PAF*(1), that is equation (2) at $t=1$. If disease prevalence (rather than an estimated incidence) is used in the argument prev, PAF_calc_discrete will estimate (1). Note that when lifetime disease incidence across the cohort is low, relative risks and hazard ratios should correspond and one would expect equation (1) to be approximately equal to (2) at varying *t*.

As described earlier, confidence intervals can be specified with ci=TRUE. The following command estimates PAF in the same way, but adds confidence intervals: 
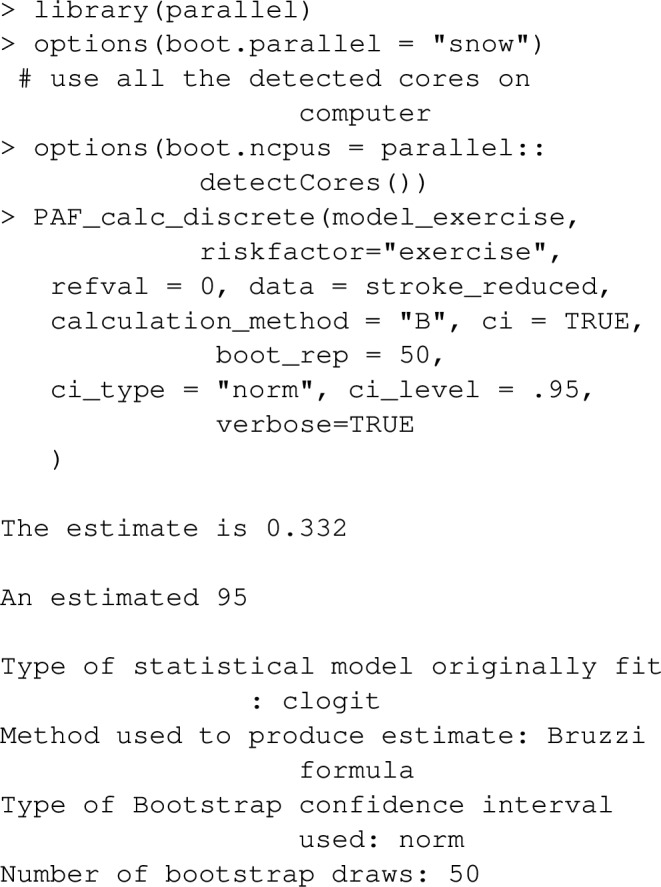
 For PAF calculations with cross sectional datasets, a glm model should first be fit that describes the relationship between risk factor and disease, conditional on covariates. Note in this case that only logistic or log-linear binomial models are permitted in graphPAF. Provided the sample is representative of the source population, standardisation (calculation_method="D") should be used, but disease prevalence, prev, no longer needs to be specified. PAF_calc_discrete will then estimate equation (1).

By default the reference level for a binary risk factor (that is a risk factor coded as 0/1) is set to 0. In the examples above, 1 codes physical inactivity. However, PAF_calc_discrete can also estimate PAF for multiple-category risk factors provided refval is set correctly.

In cohort datasets, estimation focuses on (2). graphPAF assumes the statistical model is a proportional hazards regression for time to the event, fit via the R-function coxph, from the survival package. As an example, in the dataframe stroke_reduced, time denotes a simulated survival time to some event in the stroke controls (individuals with event=0 are considered to not have experienced the event at study completion or when they left the study, and are censored). We are interested in the proportion of the events in the sub-cohort that might have been avoided, at various follow up times, if nobody in the cohort was hypertensive. The following model might be fit, which models the relative hazard of the event as a function of hypertension and possible confounders: 
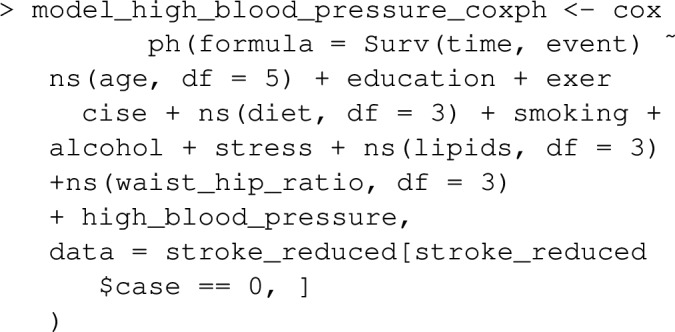
 At time 0, nobody had experienced an event, but over time the cumulative number of events, and also the proportion of events that might be avoided in the absence of the risk factor, will change. The user can specify the times, *t*, at which to calculate *PAF*(*t*) using the argument t_vector: 
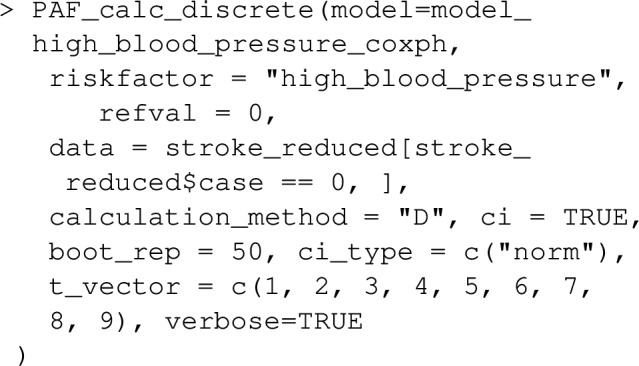

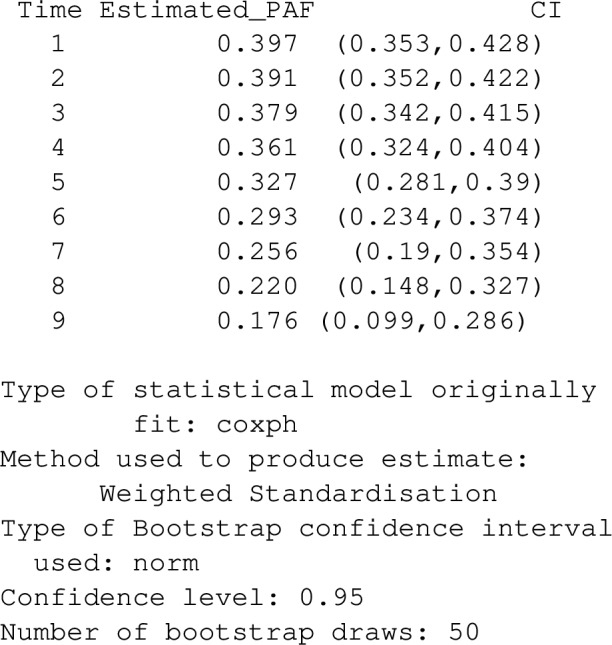
 The results indicate that while 39.7% of events that happen within a year might have been avoided in a hypertension-free population, only 17.6% of events that happen within 9 years would be avoided. This is the typical pattern one expects for an event such as death which can only be delayed but not prevented by the risk factor’s absense.

If it is preferred to estimate (3) rather than (2), and data on competing events exists, a weighted Cox model should instead be used with weights calculated using the function finegray from the survival package. Sending the weighted Cox model to PAF_calc_discrete will utilise the Fine Gray modification of (7) described earlier. See [24] for more details.

#### Estimation of PAF for data collected on surveys

PAF can be estimated with survey data by using the argument weight_vec in the PAF_calc_discrete function, where weight_vec is a vector of survey weights, that correspond to the inverse of the sampling fractions. A regression model (glm or coxph) estimated with weighted maximum likelihood with the same weights should be given as the model argument in this case. For instance, stroke_reduced, contains a column of weights, weights, giving approximate inverse sampling fractions, 0.9965 for a control and 0.0035 for a case. We can use these as illustration for how to estimate PAF from a survey. First, a glm is fit with weighted likelihood to ensure that the estimated probabilisties are representative of the population, using the inverse sampling fractions as the argument weights: 
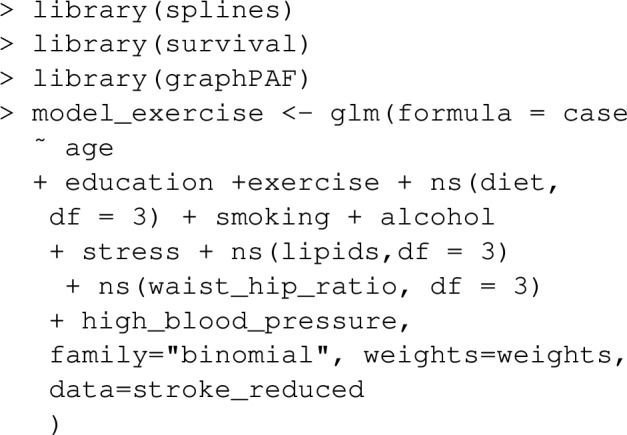
 Then PAF is estimated using calc_PAF_discrete, with the argument weight_vec set to the same vector of inverse sampling weights: 
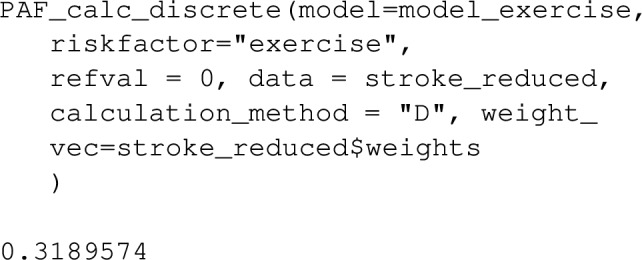
 Note that confidence intervals will be calculated using standard Bootstrap techniques (including bootstraping the inverse sampling fractions weight_vec) and may give incorrect results for surveys involving cluster sampling. One work-around would be to use graphPAF as a tool to generate point estimates, but to design a custom resampling regime that replicates the population sampling scheme used in the survey, and Bootstrap according to this custom sampling scheme.

#### Estimation of PAF using Summary data

The functions paf_miettinen and paf_levin implement estimators based on (9) and (10). If confidence intervals for the relative risk and prevalence are available, these functions will return confidence intervals for PAF based on approximate propagation of imprecision, [25].

As an example, suppose we did not have the full stroke_reduced dataset, but instead only had 95% confidence intervals for the prevalence of inactivity: (0.843,0.855), unadjusted odds ratio relating inactivity and stroke (1.514, 1.833) and the odds ratio between inactivity and stroke, adjusted according to the clogit model given earlier in this section: (1.427, 1.806). We will use these odds ratios to provide approximate estimates for the relative risks, $RR_u$ and $RR_c$ in (9) and (10). Estimates for PAF and associated confidence intervals are produced by: 
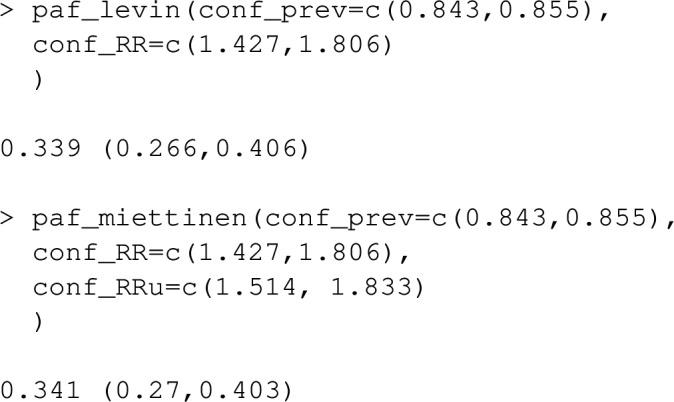
 If the risk factor has multiple non-reference levels, conf_ prev, conf_RR and conf_RRu should be specified as matrices, with each row giving separate confidence intervals for prevalence and relative risk at each non-reference level of the risk factor. In this above example, the estimated PAF and confidence interval from paf_miettinen are very similar to those produced via calc_PAF_discrete, with the default setting calculation_method="B". If the relative risk of inactivity varies substantially over strata of confounders and this effect modification is reflected by appropriate interaction terms in a statistical model, the results of the two approaches will differ, with PAF_calc_discrete being more accurate. As mentioned in the subsection “Estimates from summarised data”, Levin’s formula generates asymptotically biased estimates of PAF when $RR_c$ and $RR_u$ differ, even if the estimate of $RR_c$ substituted into the formula is correctly adjusted for confounding. Here, the extent of confounding is relatively minor, and the results of paf_miettinen and paf_levin are very similar.

### Estimation of impact fractions

While population attributable fractions (PAF) can summarise the overall impact or importance of a risk factor on disease burden, they tend to give an overly optimistic impression of what an intervention on that risk factor might achieve. The predominant reasons for this are first that it may be difficult if not impossible to eliminate the risk factor from the population (think of the difficulties in preventing all forms of smoking or alcohol-use or enticing an entire population to change their dietary habits) and second that even if one could eliminate the risk factor, disease risk in individuals who previously were exposed might not equal the disease risk if they were never exposed (for instance, former smokers may have higher disease risk than comparable individuals who never smoked) [26].

In contrast, population impact fractions purport to measure the proportional reduction in disease risk from a realistic health intervention that may reduce the prevalence of a risk factor (rather than eliminate the risk factor), or perhaps favorably change the collective statistical distribution of many risk factors. The function impact_fraction in graphPAF can estimate impact fractions under the study designs considered above (cross-sectional, cohort and case–control). We first need to specify how the health intervention changes the distribution of risk factors that might affect disease, through the new_data argument. For instance, imagine a health-intervention (perhaps a national campaign to encourage walking) reduces the prevalence of inactivity by 20%. Assuming the intervention has no effect on any other risk factor, the following code shows how such an intervention might be specified using the new_data argument 
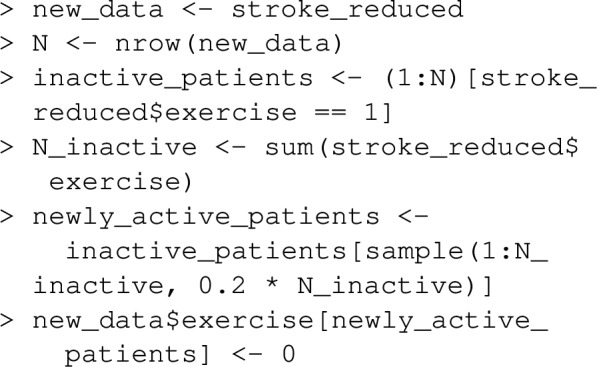
 The impact fraction for such an intervention is then calculated using: 
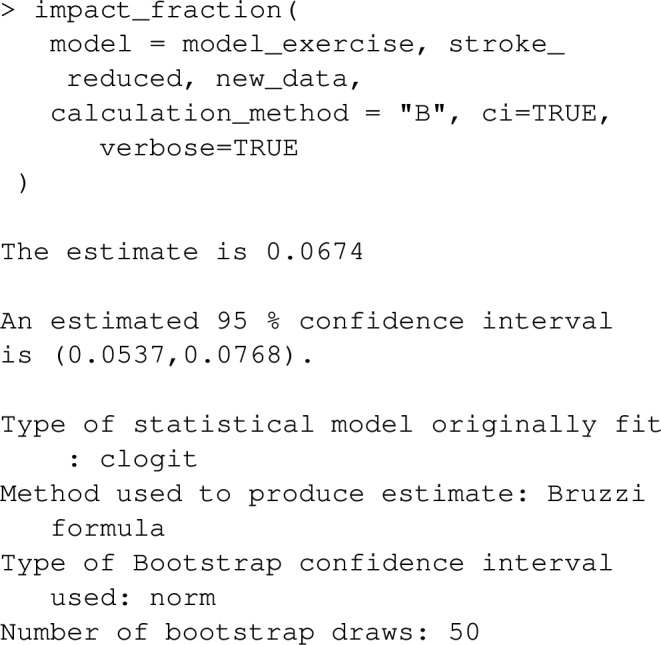
 indicating that the health intervention might result in a 6.7% reduction in the rate of strokes. Note that this calculation really refers to the difference in disease risk in two comparable populations, one with a reduced rate of inactivity. Since changing one’s behaviour may not completely eliminate cumulative damage due to prior unhealthy lifestyle, this estimated 6.7% might overestimate the impact of the intervention at least in the short term. If the 20% reduction in ‘inactivity’ is sustained through the population over years, this estimate may approximate the long run effect of the health intervention.

### PAF nomograms

**Fig. 1:**
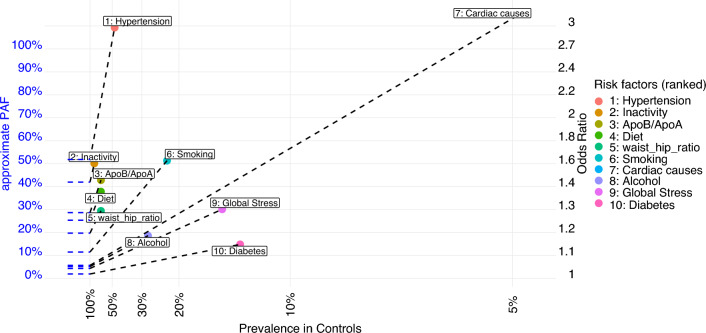
Fan Plot displaying Prevalences, odds ratios and approximate PAF for INTERSTROKE risk factors. Approximate PAF is represented as both the slope of the line adjoining a point to the y-axis, and also the y-axis intercept of that adjoining line. The fan plot indicates that hypertension and inactivity are the two most prominent risk factors in stroke pathogenesis. Cardiac disease is an outlier on the plot. While it has the highest estimated relative risk, it has low prevalence (less than 5%) in comparison with the other risk factors and is only ranked 7th in terms of disease burden

graphPAF facilitates plotting of the inter-relationships between prevalence, odds ratios and attributable fractions over multiple risk factors using methods described in detail in [7]. These plots utilise the concept of ‘approximate-PAF’, derived in the same paper:

11 $$\begin{aligned} PAF_{approx} = log(OR) \pi _{control} \approx PAF \end{aligned}$$

where *OR* is the causal odds ratio between a risk factor and disease, and $\pi _{control}$ is the prevalence of the risk factor in controls. This approximation stems from a Taylor expansion of the PAF around a relative-risk of 1, and will be most accurate for risk factors that have relatively small effects on a relatively rare outcome. One interesting observation regarding approximate PAF is the symmetric roles that risk factor prevalence and log-odds ratio play in its definition; indicating that similar changes in either lead to a similar impact on disease on a population level. To create a fan plot, risk factor data (names, prevalences and log-odds ratios) must be first summarised into an rf_summary object before plotting. For instance: 
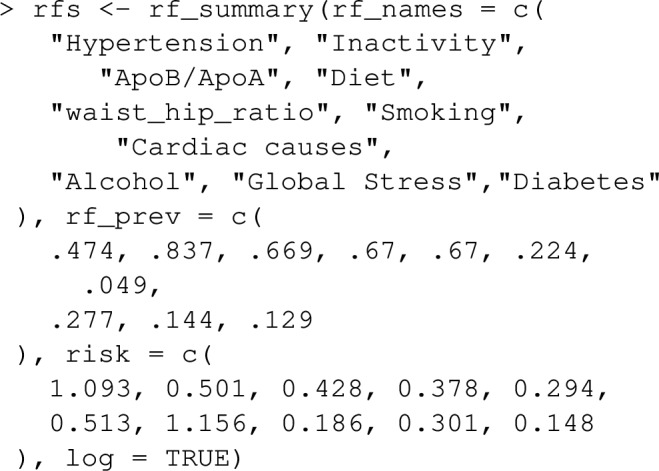
 creates such an object for 10 risk factors from the INTERSTROKE database. rf_prev represents the prevalence of the risk factor in controls. For risk factors with more than 2-levels (here ApoB/ApoA, waist_hip_ratio and alcohol have 3 levels), the prevalence of the non-reference levels of the risk factor should be used as rf_prev. While technically, rf_prev should be the prevalence of the risk factor in controls, this can be substituted with population-prevalence when prevalence in controls is unavailable if the disease is rare. By default, the argument risk should specify confounder-adjusted log-odds ratios for association between risk factor and outcome, although odds ratios or risk ratios can be used via the setting log=FALSE. Note that log-odds ratios can be conveniently estimated via logistic regression models. Plotting this rf_summary object, using default settings, produces Fig. 1 below. 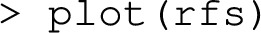

Approximate PAF is represented on a fan plot as both the slope of the line adjoining a point to the y-axis, and also the y-axis intercept of that adjoining line. Fan plots are read clockwise from the upper left corner along the rays of decreasing approximate PAF (which is again the slope of the ray), and display risk factor prevalence and odds ratio (based on the x-axis and y-axis intercept of a particular point) for the risk factors under comparison, in addition to the approximate PAF.

**Fig. 2:**
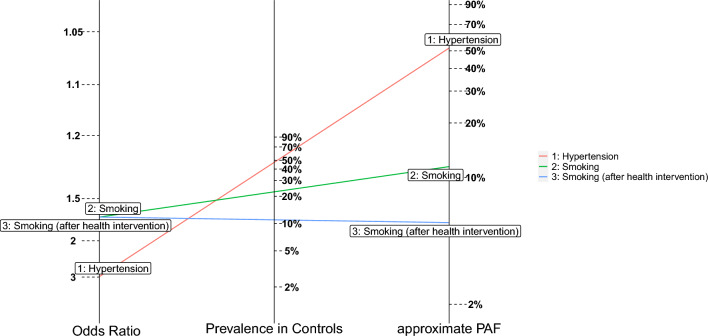
Attributable fraction nomogram for INTERSTROKE risk factors. Nomograms like the above give an alternative way to visualise the relationships between odds ratios, prevalences and approximate PAF. They can also be used to visualise interventions. For instance, the green and blue lines represent smoking in a population pre and post-intervention. The odds ratio for smoking isn’t affected by the intervention, but the prevalence is. The effect of the intervention for smoking PAF can be visualised by rotating the line for smoking (using the left axis odds ratio as a pivot) through the new prevalence post-intervention

Imagine now a successful health intervention that reduces the prevalence of smoking by about 50%. This information might be displayed in a rf_summary object as follows: 
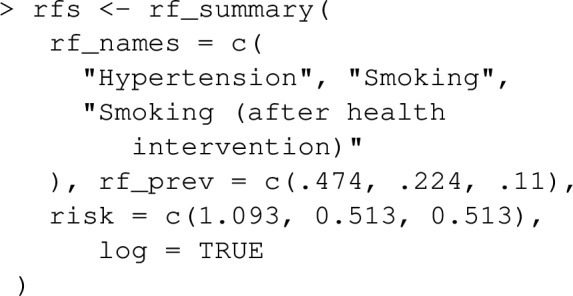
 Like a fan plot, attributable fraction nomograms display joint information on prevalence, odds ratio and approximate PAF, but this time on three vertical axes, with a risk factor represented by the line connecting these three data-points. An intervention will usually work by changing the population prevalence of the risk factor, without affecting the odds ratio. This can be graphically represented by rotating the line for the risk factor, using the (unaffected) odds ratio as a pivot, from the old prevalence through the new prevalence, as Fig. 2 represents. Of course, other risk factors can also be represented on this plot (as is hypertension here). Plotting a rf_summary object with argument type="rn" produces the Figure below. If preferred, using type = "n", uses the odds ratio, rather than prevalence as the center-axis, with risk factor prevalence being the left-hand axis, but is otherwise interpreted similarly. 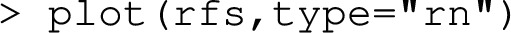

## Estimation with continuous exposures

Frequently, a discrete risk factor such as hypertension is generated by the truncating an underlying continuous exposure, such as blood pressure. Not accounting for this underlying continuity may result in underestimation of disease burden attributable to the exposure. This is because individuals categorised within the lower of two exposure categories may still be still at elevated risk of disease. As an example, suppose hypertensive is defined as systolic blood pressure above 140 mm/Hg. Then an individual with systolic blood pressure of 139 would fall into the ‘reference’ group, but might have some increased risk of cardiovascular disease compared to if their blood pressure was 120. [12] discusses these issues and suggests a variety of appropriate estimands for continuous exposures.

### Estimands for PAF with continuous exposures

Using the notation from [12], we consider the exposure for a randomly selected individual from the population as a continuous random variable, *X*, with *Y* representing a binary disease outcome. We let $Y_{x}$ represent the potential outcome if $X=x$, which we assume is well defined. Assuming that $P(Y_{x}=1)$, considered as a function of *x*, has some minimum value $x_{min}$ within the physiological limits of the exposure *X*, we define *PAF* as:

12 $$\begin{aligned} PAF=\frac{P(Y=1)-P(Y_{x_{min}}=1)}{P(Y=1)}, \end{aligned}$$

**Fig. 3:**
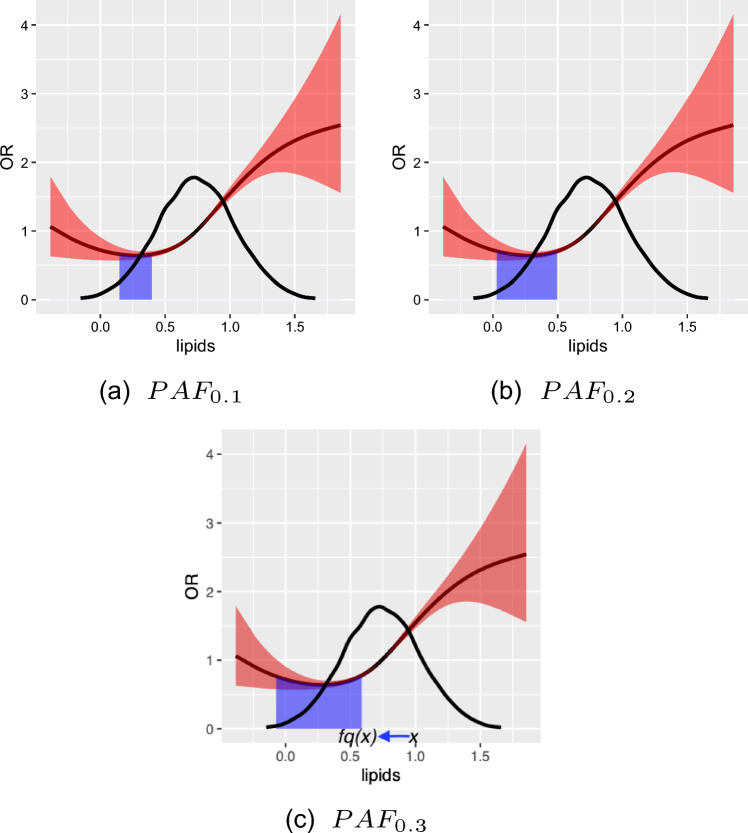
Estimated effects of blood lipid levels on the OR of stroke. The density of lipids and pointwise 95% confidence bands for the odds ratios are also plotted. Also shaded blue are the target regions for the intervention associated with $PAF_q$ for various *q*. For instance $PAF_{0.1}$ corresponds to the smallest 10% of risk

As explained in [12], the estimand (12) may be difficult to estimate when $x_{min}$ falls in the extremes of the exposure distribution since it will require estimating probabilities of disease or relative risks at $x_{min}$; these are possibly extreme extrapolations of the fitted model. As an alternative, the family of estimands: $PAF_q$ for $q \in (0,1)$ are suggested in [12] as alternative metrics. $PAF_q$ is the impact fraction for an intervention that changes the exposure only for the 100(1-q)% of individuals who have the most hazardous values of exposure. Suppose an individual, who is affected by this intervention, has exposure value *x*. The intervention shifts their exposure to the value $f_q(x)$, closest to *x*, from the set of physiologically possible exposure values having the lowest 100q% of risk. For instance, the intervention corresponding to $PAF_{0.3}$ for the variable, lipids in stroke_reduced is illustrated by the region shaded blue, in the bottom panel of Fig. 3. An individual with exposure value $x=1$ is affected by the intervention. Their exposure value is shifted to the closest value, $f_{0.3}(1)=0.59$ that is within the lowest 30% of risk, noting that the density curve in the figure describes the distribution of physiologically possible exposure values. Individuals who have exposure values in the blue shaded region are unaffected by the intervention.

Estimating $PAF_q$ for $q \ge 0.1$ will often involve less extrapolation than (12), and consequently estimators of $PAF_q$ are likely to have lower variance. $PAF_q$ also has a more concrete real world interpretation as the impact fraction for an achievable intervention. In practice, we may want to compare estimated values of $PAF_q$ across a range of reasonable values for *q*.

$PAF_q$ is defined more precisely as:

13 $$\begin{aligned}{} & {} PAF_{q}\nonumber \\ {}{} & {} =\frac{P(Y=1)-P(I\{X\in R_{q}\}Y+I\{X\notin R_{q}\}Y^{f_{q}(X)}=1\}}{P(Y=1)} \nonumber \\ \end{aligned}$$

where $R_{q}$ is the interval of exposure values corresponding to the bottom 100*q*% of risk and $f_{q}(X)$ is the closest point in the closure of $R_{q}$ to *X*. Note that as $q \downarrow 0$, $PAF_q \uparrow PAF$.

Under continuous analogs of the conditions 1), 2) and 3) listed on pages 5 and 6, (13) can be estimated as

14 $$\begin{aligned} {\hat{PAF}}_{q}{=}\frac{\sum _{i{=}1}^N I\{X_i\notin R_{q}\}(\widehat{P(Y{=}1 \mid X_i,C_i)}{-}{\hat{P}}(Y{=}1 \mid \,{\hat{f}}_{q}(X_i),C_i))}{\sum _{i{=}1}^N{Y_i}},\nonumber \\ \end{aligned}$$

15 $$\begin{aligned} {\hat{PAF}}_{q}{=}1{-}\frac{1}{N_c}\sum _{i \le N: Y_i{=}1}I\{X_i\notin R_{q}\}\frac{{\hat{P}}(Y{=}1 \mid \,{\hat{f}}_{q}(X_i),C_i)}{\widehat{P(Y=1 \mid X_i,C_i)}}, \nonumber \\ \end{aligned}$$

 and

16 $$\begin{aligned} \hat{PAF_q(t)} = \frac{\sum _{i=1}^N{(e^{-\hat{H}_0(t)\hat{h}(C_i,\hat{f}_q(X_i)}-e^{-\hat{H}_0(t)\hat{h}(C_i,X_i)}})}{\sum _{i=1}^N{(1-e^{-\hat{H}_0(t)\hat{h}(C_i,X_i)})}} , \nonumber \\ \end{aligned}$$

respectively for cross sectional, case control and cohort designs, where $N_c=\sum _{i=1}^N Y_i$ and ${\hat{f}}_{q}(x)$ the estimated value for $f_{q}(x)$ and $\widehat{P(Y=1 \mid x,c)}$, the estimated probability of disease, when the risk factor is *x* and the covariates are *c*.

### Estimation using PAF_calc_continuous

Here we consider the convenient case where a group of continuous risk factors: waist_hip_ratio, diet and lipids all have the same set of underlying confounders, and subsequently estimated effects of each risk factor can be obtained from a single statistical model. The following code demonstrates how such a model might be specified for a case–control dataset. Note that the continuous exposures waist_hip_ratio, diet and lipids appear in the model as natural spline terms. 
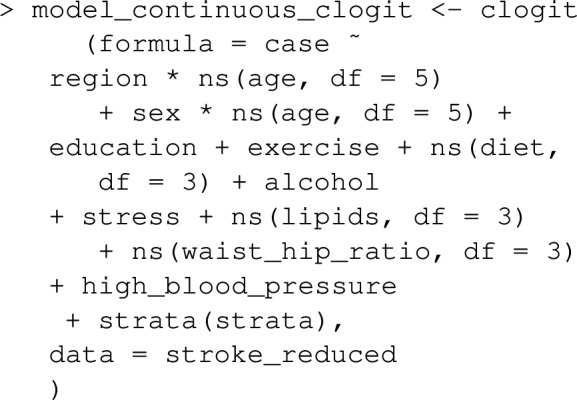
 Having fit the model, the function PAF_calc_conti nuous estimates $PAF_q$ at any desired set of quantiles, q_vec, by implementing equations (14), (15) and (16). One call to PAF_calc_continuous can estimate $PAF_q$ for a number of exposures, by using the argument riskfactor _vec. The resulting object is essentially a dataframe with rows for each (risk factor, $PAF_q$) combination and columns corresponding to quantiles which can be printed and plotted as follows: 
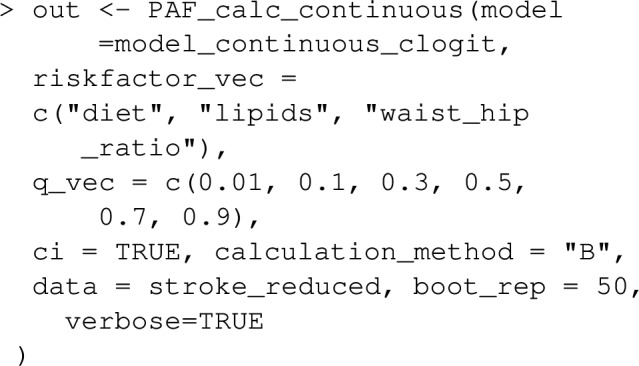

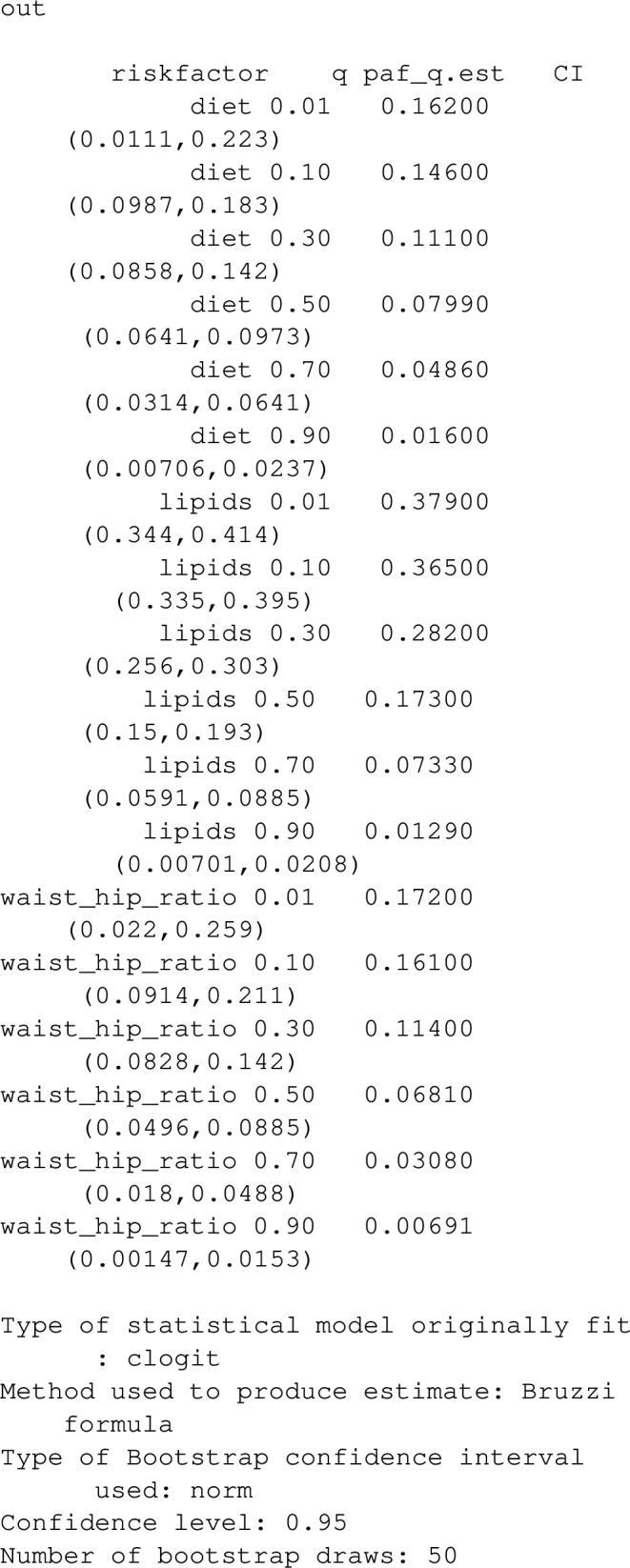


Using the argument "calculation_method="B", as above uses (15) to estimate $PAF_q$ (appropriate in case–control designs), in contrast, "calculation_method ="D" uses (14), whereas (16) will be used for proportional hazard models. Note that in the case of a survival response, $PAF_q(t)$ can only be evaluated at a single time, t, specified as t_vector being a single element.

### Plots

One can evaluate the estimated shape of the exposure/outcome relationship, and visualise the interventions corresponding to a particular value of $PAF_q$ using the function plot_continuous. As an example: 
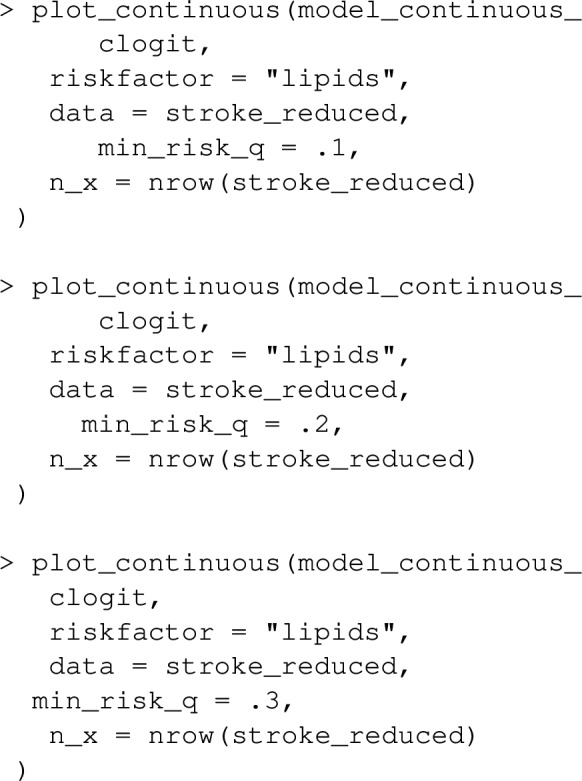
 produce estimated relationships between lipids and OR of stroke (with the median value for lipids as a reference by default), in addition to highlighting the exposure-ranges for the interventions corresponding to $PAF_{0.1}$,$PAF_{0.2}$ and $PAF_{0.3}$. To enable assessment of the physiologically possible values for the exposure, the estimated population density of exposure values is included in the plot.

**Fig. 4:**
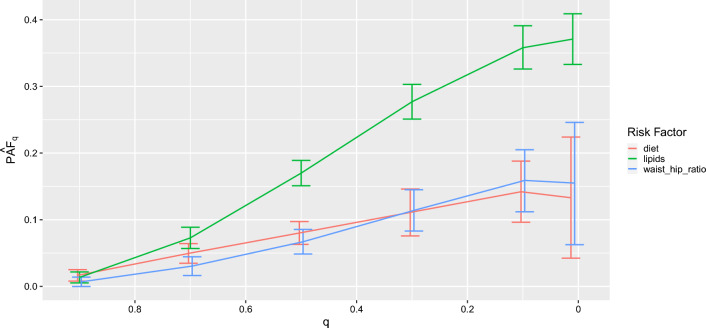
plotting $PAF_q$ over multiple risk factors. The figure indicates that comparable interventions on diet and waist hip ratio (for instance intervening on the 50% of most hazardous exposure values in the population as is the case in $PAF_{0.5}$ may have similar effects for diet and waist hip ratio, but much larger effects for lipids). As can be seen in the plot, the confidence intervals for $PAF_q, q \ge 0.1$ are narrower than the confidence interval for $PAF_{0.01} \approx PAF$, reflecting the fact that $PAF_q$ is easier to estimate than *PAF*

$PAF_q$ can be plotted against *q* for several risk factors by simply plotting the returned object from a call to PAF_calc_continuous, as seen in Fig. 4. Such a plot allows one to visually assess the relative benefits of comparable and achievable interventions on differing risk factors. 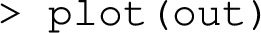

The results in Fig. 4 indicate that comparable interventions targeting waist hip ratio and diet may have similar effects on disease burden, with interventions on lipids having larger effects. This might motivate an intervention on lipid levels (for example, increased statin use when appropriate) over interventions on diet or BMI, although admittedly many other factors may dictate what if any intervention may be chosen in practice.

## Pathway-specific PAF calculations

While PAF provides an overall measure of the importance of a particular disease risk factor in causing disease on a population level, the mechanisms by which the risk factor effects disease may also be of interest. For instance, perhaps physical inactivity increases blood pressure which subsequently increases the risk of stroke. Alternatively, physical inactivity might indirectly increase the risk of stroke through weight gain or increased cholesterol levels. In this context, the variables blood pressure, weight gain and cholesterol are regarded as ‘mediators’, that is they are intermediate variables on differing causal pathways each partially explaining the causal relationship between inactivity and stroke. How important might each pathway be in disease pathogenesis? In [10], this question is addressed by defining an attributable fraction for a particular mediating pathway. Roughly this ‘pathway-specific’ attributable fraction (PS-PAF for short) can be interpreted as the relative decrease in disease prevalence if a particular mediating pathway didn’t exist. For instance imagine there was no effect of physical inactivity on blood pressure; what percentage of stroke might be avoided in such a population? Letting $M^1,\ldots ,M^K$ represent *K* known mediators of the risk factor outcome relationship, $A \in \{0,1\}$ a risk factor and $Y \in \{0,1\}$ a disease outcome, the PS-PAF for mediator $k \le K$ is denoted as:

17 $$\begin{aligned} PAF_{A->M^k->Y} = \frac{P(Y=1)-P(Y_{A,M_{0}^k}=1)}{P(Y=1)} \end{aligned}$$

$P(Y_{A,M^k_{0}}=1)$ can be interpreted as disease prevalence in a hypothetical population which mirrors the actual population in the values of the risk factor *A*, but where the values for mediator, $M^k$, behave as if the risk factor didn’t exist (note that on an individual level $M^k_{0}$ is the potential outcome for the $k^{th}$ mediator assuming no exposure to the risk factor, that is $A=0$). As described in [10], interpretations for pathway-specific attributable fractions subtly differ based on the causal identifiability assumptions assumed. We are describing the mechanistic interpretation here, although two other interpretations exist. We won’t go into these details here and instead refer the interested reader to [10].

In addition to pathway specific PAF for indirect pathways, one can also define an attributable fraction for all ‘unobserved’ or unknown pathways:

18 $$\begin{aligned} PAF_{A->Y} = \frac{P(Y=1)-P(Y_{0,M^1,\ldots ,M^K}=1)}{P(Y=1)} \end{aligned}$$

(18) denotes the ‘direct’ pathway specific population attributable fraction, and represents the contribution of mechanisms by which the risk factor affects disease, other than those represented by pathways through $M^1$,....,$M^K$ (Note that $P(Y_{0,M^1,\ldots ,M^K}=1)$ can be interpreted as the disease prevalence in a population where the risk factor was eliminated but with the joint distribution of mediators $M^1,\ldots ,M^K$ being unaffected). Under the assumptions listed in [10] (with the additional assumption that mediators are on separate causal pathways between the risk ractor and disease), estimating (17) requires fitting a model for the mediator $M^k$ conditional on both the risk factor, *A*, and the confounder-vector for the exposure outcome relationship, *C* (Note that these models estimate $P(M^k=m \mid A,C)$ for a discrete mediator and $E(M^k \mid A,C)$ for a continuous mediator), in addition to fitting a model for the disease outcome, *Y*, conditional on the exposure *A*, mediators $M^1,\ldots ,M^K$ and the same set of confounders *C*. (This second model estimates $P(Y=1 \mid A,C,M^1,\ldots ,M^K)$). When $M^k$ is continuous, the following estimator for (17) is used:

19 $$\begin{aligned}{} & {} \widehat{PAF}_{A->M^k->Y}\nonumber \\{} & {} =\frac{\sum _{i = 1}^{N}{w_iY_i}-\sum _{i = 1}^{N}{w_i{\hat{P}}(Y=1 \mid A_i,C_i,{\hat{M}}^k_i, \mathbf {M_i^{\ne k})}}}{\sum _{i = 1}^{N}{w_iY_i}} \nonumber \\ \end{aligned}$$

with ${\hat{M}}^k_i$ = $M^k_i - \widehat{E(M^k \mid A=0,C_i)}$, with $C_i$ and $M^k_i$ representing the observed values of the confounder vector and $k^{th}$ mediator for person *i*, and $\mathbf {M_i^{\ne k}}$, the observed values for other mediators for the same individual. Weights $w_i$ are used to account for possible case–control structure. For representative cross sectional samples, these weights should be set to 1 (the default). In contrast, for case control data, these weights can be set based on estimated disease prevalence. In the case that the mediator $M^k$ is discrete, having possible values given by the set $\mathcal {M}^k$, a slightly different estimator is used:

20 $$\begin{aligned} \begin{aligned} \widehat{PAF}_{A->M^k->Y}=\frac{\sum _{i = 1}^{N}{w_i(Y_i - \sum _{m \in \mathcal {M}^k}{\widehat{P(M^k=m \mid A_i=0,C_i)}}{\hat{P}}(Y=1 \mid A_i,C_i,M^k=m,\mathbf {M_i^{\ne k})}}}{\sum _{i = 1}^{N}{w_iY_i}} \end{aligned} \end{aligned}$$

The direct PS-PAF is slightly easier to estimate, as one only needs to fit the outcome model that conditions on the risk factor, *A*, covariates *C* and mediators, $M^1$,...,$M^K$:

21 $$\begin{aligned} \widehat{PAF}_{A->Y}=\frac{\sum _{i = 1}^{N}{w_iY_i}-\sum _{i = 1}^{N}{w_i\widehat{P(Y=1 \mid A_i=0,C_i,M^1_i,\ldots ,M^K_i)}}}{\sum _{i = 1}^{N}{w_iY_i}} \end{aligned}$$

**Fig. 5:**
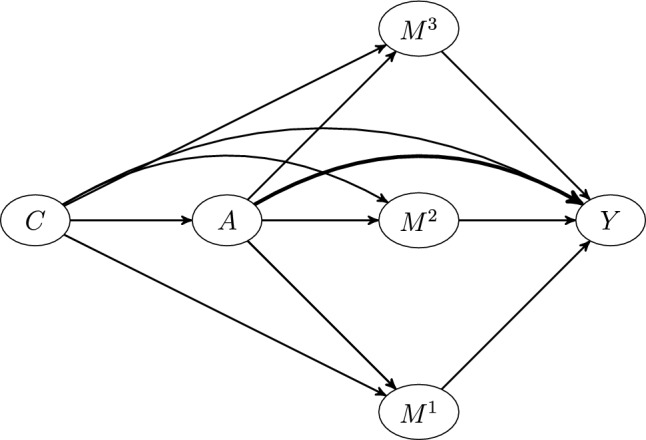
Mediators on separate causal pathways. $M^1$, $M^2$ and $M^3$ mediate the causal relationship between *A* and *Y*. These mediators represent independent mechanisms by which *A* affects *Y* in that any pathway of direct arrows originating from *A* and ending at *Y* can only involve one of the three mediators

### Examples

To illustrate these calculations with graphPAF, suppose we wish to estimate pathway-specific attributable fractions for the 4 pathways from physical inactivity to stroke through waist hip ratio, through blood lipid counts, through high-blood pressure, and through any mediating pathways other than waist hip ratio, blood pressure and lipids from the simulated dataset stroke_reduced included in the graphPAF package. The assumed causal structure is represented by the DAG in Fig. 5. Since stroke_reduced is a case control dataset, weighted models for each mediator and the response need to be fit, to replicate the fits one would expect from a representative sample of the population. In stroke_reduced, these weights are already in the dataset and are based on an average incidence of 0.0035 new strokes per person per year. If not directly available, the weights vector can be calculated using the function data_clean. For instance, if we instead thought that 0.01 was the correct incidence, we could use 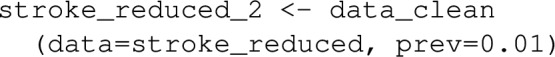


A column of weights, appropriate to estimate probabilities of disease when the prevalence is 0.01, is then included in the dataframe stroke_reduced_2. As a default, data_clean returns a data frame having the same number of columns as the input dataset, but removes rows having at least one missing value. If only a subset of columns from the dataset are required, these can be specified through the vars argument.

Having calculated these weights, models for the response and a list of models for the mediators can be specified: 
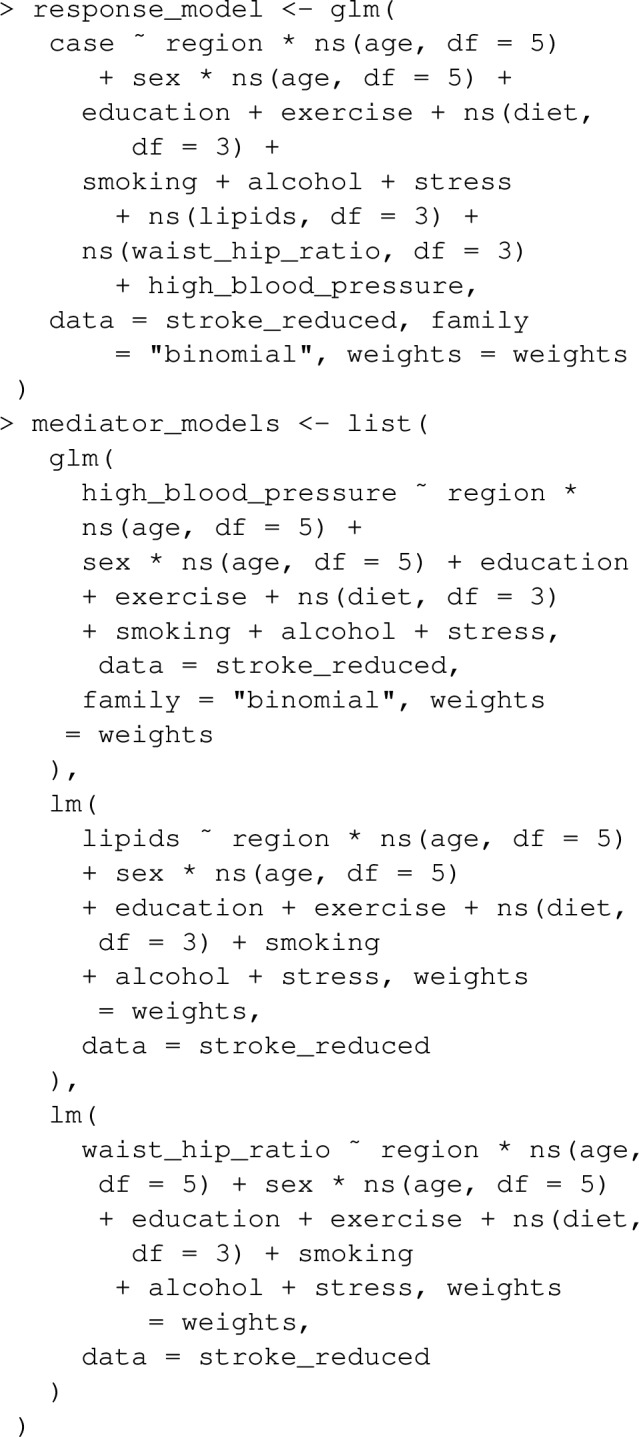


The response model and the list of mediator models is then sent to ps_paf, which implements the estimators: (19),(20) or (21) with the fitted models. Again, for case control datasets, the argument prev needs to be specified for correct calculation of the weights. 
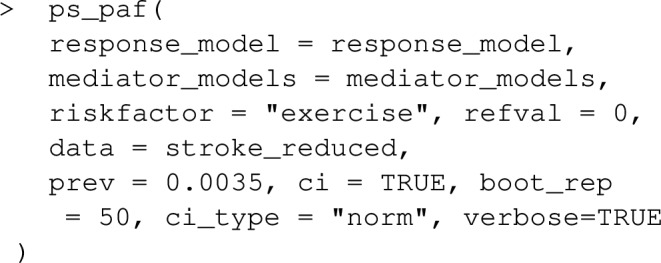

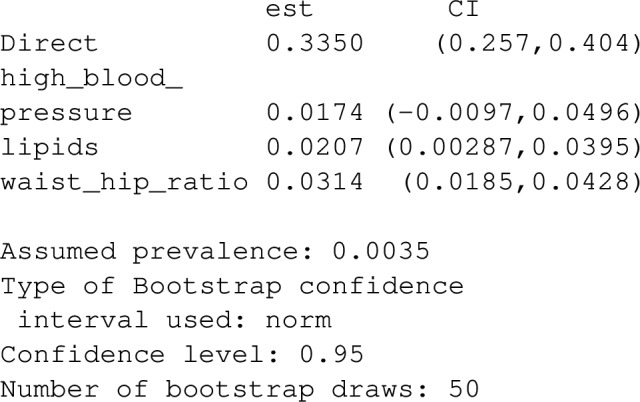
 The results indicate that only a small proportion of the disease burden due to physical inactivity is attributable to pathways involving lipids, blood pressure and waist hip ratio. For instance, if the pathway from physical inactivity to stroke through waist hip ratio were disabled (in that physical inactivity had no deleterious affect on waist hip ratio), relative stroke prevalence would only decrease by 3.1%, with similar interpretations and small PS-PAFs for the pathways through lipids and high blood pressure.

**Fig. 6:**
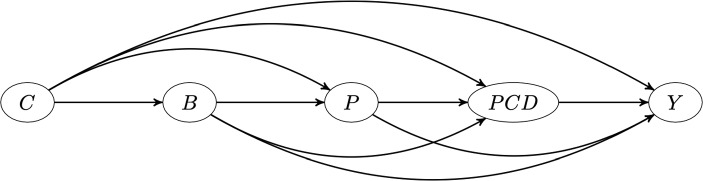
DAG showing causal structure assumed for the applied examples in this manuscript. For the simulated INTERSTROKE dataset, we assume that each node represents multiple risk factors as follows: C represents the Confounders (age,region,sex and education), B represents Behavioural risk factors: (exercise, alcohol use, smoking, stress levels and diet), P represents risk factors indicating physiology: (blood pressure, blood lipids and waist hip ratio), PCD represents pre-clinical disease: (diabetes and cardiac symptoms such as atrial fibrilation). Y is a 0/1 indicator for stroke occurance

## Joint PAF

Joint attributable fractions refer to the collective disease burden that can be appropriated to a collection of risk factors. For instance the INTERSTROKE study [22] estimates that roughly 90 % of incident strokes might be avoided if 10 major modifiable stroke risk factors were removed from the population. More formally, the joint population attributable fraction for a set of risk factors, ${\textbf{S}}$ can be defined as:

22 $$\begin{aligned} PAF_{{\textbf{S}}} =\frac{P(Y=1)-P(Y(\mathbf {0_{S}})=1)}{P(Y=1)}, \end{aligned}$$

with the shorthand: $Y(\mathbf {0_{S}})$ representing the potential outcome where the subset of risk factors ${\textbf{S}}$ have been set to their reference levels. Traditionally, such calculations were performed via multivariable regression models that include the set of variables that are to be eliminated. For instance to estimate a joint PAF for stroke associated with stress and a diagnosis of diabetes, disease risk in the data-collected might be compared to predicted disease risk if diabetes status and stress were set to their reference levels, with the predicted disease risk being computed via a single fitted logistic model. While this approach may be fine if diabetes status and stress share the same set of confounding variables (proviso that the model for stroke risk includes these confounders and is correctly specified) bias may result when effects of one of the risk factors confounds the relationship between the response and other risk factors of interest. This is the case here as blood pressure, which is an effect of stress according to Fig. 6, confounds the relationship between diabetes and stroke. For these kinds of causal structures, while predicted risks derived via a single regression may correctly reflect the probability that an individual in the dataset has disease, *conditional* on their having reference values for the risk factors under investigation, they will not reflect the probability of disease in the population if all individuals had reference levels for those same risk factors. In other words, the associated estimated joint PAF will not have a causal interpretation.

[9] describes how the intervention corresponding to a joint population attributable fraction (the intervention being the ‘elimination’ of a subset of risk factors) can be conceptualised via recursive application of Pearl’s do-operator [27] on the true causal graph (assumed to be a directed acyclic graph or DAG), linking risk factors, outcome and associated risk factor/outcome confounders. This observation facilitates asymptotically unbiased estimation of joint attributable fractions under general causal structures. To achieve this in practice, we need to first know the causal DAG, second have collected data on individuals $i = 1,\ldots .,N$ for all variables represented in the DAG, and finally correctly specify and fit statistical models linking each node in the causal DAG to all of its direct causes (the direct causes being those variables with arrows pointing to the node of interest). Having done this, one can use these fitted models to simulate from the joint-distribution of all variables in the graph (confounders, risk factors and outcome) corresponding to each application of the do-operator. For each application of the do-operator (corresponding to a population level elimination of a single risk factor), this simulation is itself recursive. For instance, if smoking is eliminated, smoking is first set to its reference level (no smoking) for all individuals in the current simulated dataset. Values for the direct effects of smoking (that is the nodes for which smoking is a parent in the causal graph) are then simulated from the conditional distribution of these variables assuming no smoking. Suppose blood pressure is one of the effects of smoking. Next the direct effects of variables such as blood pressure are simulated, conditional on prior simulated values for blood pressure and the other direct effects of smoking. This process (simulations of a particular node being made conditional on the simulated values for parent nodes) is continued until the response node is simulated. More details are given in [9].

Suppose then that upon elimination of a subset ${\textbf{S}}$ of risk factors, the population distribution of all variables in the causal graph is $\mathbf {P_{S}}$, and via the recursive algorithm above, we have simulated new data $\mathbf {D_{S}}$ for all variables in the causal graph (excluding the response) under $\mathbf {P_{S}}$. Our estimate for (22) is then:

23 $$\begin{aligned} PAF_{{\textbf{S}}}=\frac{\sum _{i = 1}^{N}[w_iY_i-w_i\widehat{P(Y_i=1 \mid \mathbf {D_{S}}})]}{\sum _{i = 1}^{N}w_iY_i}, \end{aligned}$$

where ${\hat{P}}(Y_i=1 \mid \mathbf {D_S})$ represents the estimated probability of disease for individual *i* under the simulated data structure for risk factors and confounders represented by $\mathbf {D_S}$ (this probability depends on $\mathbf {D_S}$ through the simulated values for individual *i* at those risk factors and covariates that are assumed to directly affect the outcome). This approach can be applied to cross-sectional and case–control datasets, where as before the argument prev is utilised to change the weighting in case–control datasets. Note that the above estimator may be randomised, that is estimating joint PAF twice using the same data may give slightly different results, since differing simulated datasets $\mathbf {D_S}$ will likely be used in (23) on each ocassion. The degree of randomization in the resulting estimator will generally be small for large datasets, although if desired the estimator (23) can be averaged over several independently simulated versions of $\mathbf {D_S}$ to reduce variability. In some cases, $\mathbf {D_S}$ may not vary over differing simulations. For instance, for reasons described in [10], continuous variables in $\mathbf {D_S}$ are simulated by adding model predicted residuals to the predicted values given the current values of their parents. As a result, randomness in $\mathbf {D_S}$ can only be generated by discrete risk factors or confounders that are graph-descendants of risk factors that are eliminated.

### Data examples

The joint_paf function in graphPAF implements the procedure described above. As an example, suppose we are interested in estimating the joint PAF for stroke due to stress and blood pressure. First we need to specify the causal graph linking stress, blood pressure and stroke. In doing this, one must ensure that the confounders of any two nodes in the graph are also specified: for instance, any joint causes of stress and blood pressure must also be included. In Fig. 5, we illustrate our assumed causal structure for INTERSTROKE risk factors, which includes many confounders and risk factors other than stress and blood pressure. However, in the context of this estimation problem (and assuming Fig. 5 is correct), we can give graphPAF a reduced causal structure: we actually don’t need to specify pre-clinical disease variables PCD or physiology variables P, other than blood pressure, since they are not common causes of the target risk factors: (stress, blood pressure and stroke). In graphPAF we specify the causal graph with a list of the parents of all relevant variables in the graph as the argument parent_list, together with a vector of variable names, corresponding to the nodes of the graph, as the argument node_vec. When doing this it is important that node_vec and parent_list are in the same order. In addition, node_vec should be ordered so that parent nodes (that is causes) are positioned in the vector before their children (that is their effects). 
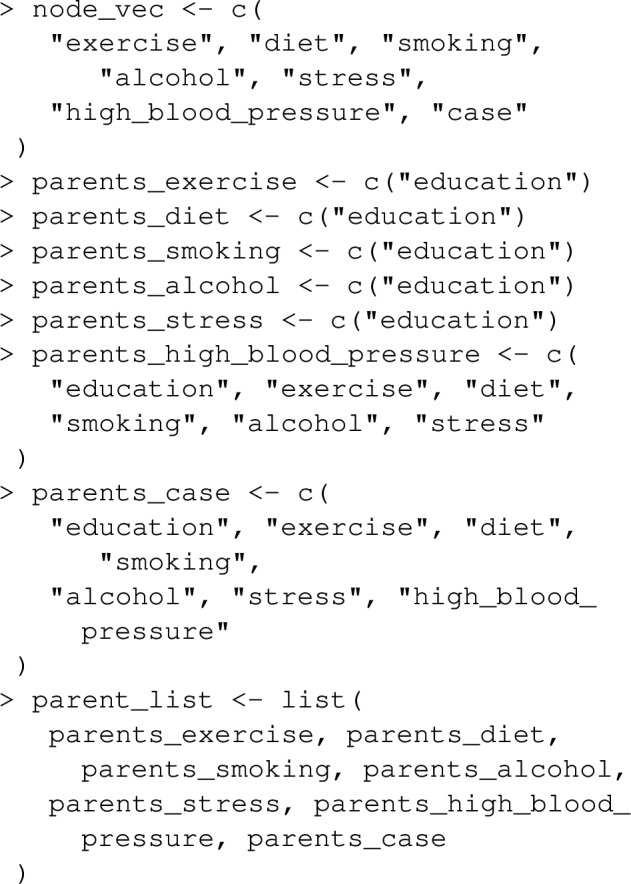
 Next, models for each variable (each time conditioning on its parents) need to be fit. In the context of joint PAF (as well as the sequential and average attributable fractions detailed in the following section), graphPAF supports simulation from linear models (fit using lm), logistic models (fit using glm) and ordinal logistic models (fit using polr from the R package MASS). Given that specification of multiple models can be time-consuming, graphPAF has a function automatic_fit that automatically fits additive models for each node in node_vec, conditioned on the parents of that node. This function can also fit non-linear relationships for continuous riskfactors or confounders using the spline_nodes argument. In the code below, diet is assumed to have a non-linear effect. Common interactions between variables that appear in all of the models can be specified by the argument common. However, in reality some of these models may require individual specification of interactions, in which case the models must be fit separately with either lm, glm or polr, before populating model_list. For case–control datasets, these models need to be fit with appropriate weighting (so that the weighted dataset set could be regarded as a representative sample) as described earlier. If automatic_fit is used, this can again be achieved automatically by specifying specifying the prev argument. As mentioned earlier, appropriate weights can also be calculated by passing the original dataset through data_clean before model fitting. 
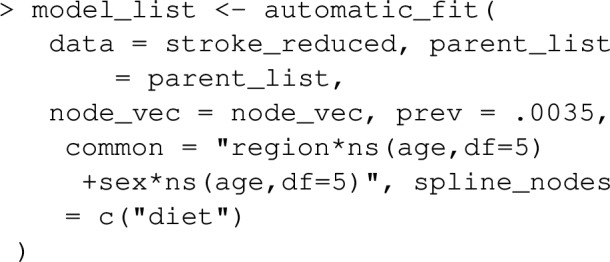
 Once model_list is specified, it can be passed to joint_paf for estimating joint PAF. Below, we compare estimated single risk factor attributable fractions for smoking and blood pressure to the joint attributable fraction for both smoking and blood pressure together. Note that the estimated joint attributable fraction (0.375) is slightly less than the sum of individual attributable fractions $(0.113+0.269=0.382)$. This is actually expected [28]: as some of the disease cases that might be prevented in a population where nobody smokes would equally be prevented in a population where nobody was hypertensive. As mentioned earlier, joint_paf can average the estimator (23) over multiple independently estimated datasets using the argument nsim. However, since no discrete graph-descendants of smoking (other than high_blood_pressure) are specified in the causal graph specified in joint_paf, $\mathbf {D_S}$ will not vary over differing simulation iterates in this example. 
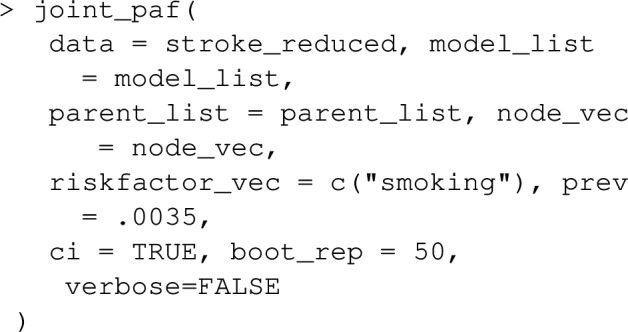

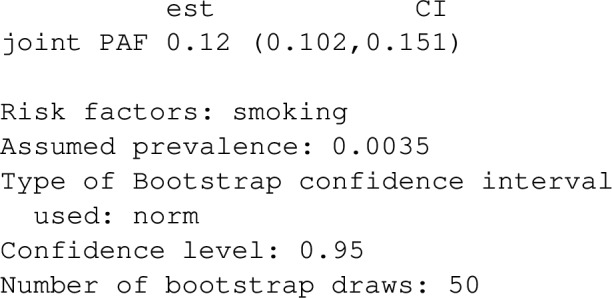

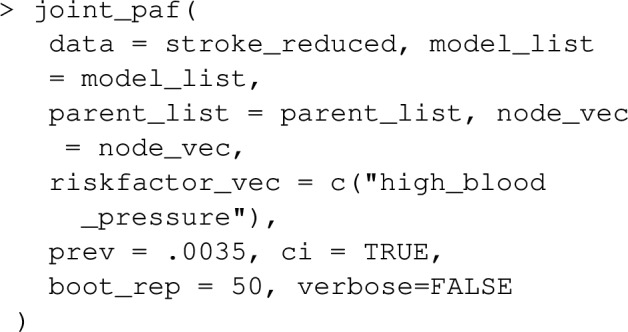

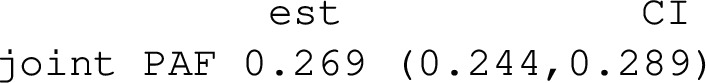

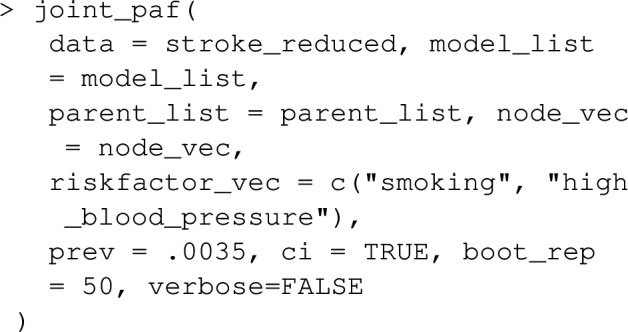

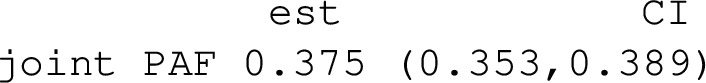


## Sequential and average PAF

Sequential attributable fractions (SAF), first described by [23] are closely related to joint attributable fractions as discussed in the previous section. They pertain to the incremental disease burden attributable to a risk factor (or more specificially to the removal of that risk factor from the population) in a population where a subset of risk factors have already been eliminated. Suppose that we number disease risk factors under consideration as: $\{1,\ldots ,K\}$. We can define the sequential PAF for eliminating risk factor $j \le K$, conditional on the subset of risk factors ${\textbf{S}} \subset \{1,\ldots ,K\}{\setminus } \{j\}$ already having being removed from the population, as the difference in joint PAF pertaining to removing $\mathbf {S \cup \{j\}}$ and the PAF pertaining to removing ${\textbf{S}}$ alone:

24 $$\begin{aligned} PAF_{j\mid {\textbf{S}}} = PAF_{{\textbf{S}} \cup \{j\}}-PAF_{{\textbf{S}}} \end{aligned}$$

Given this link between joint and sequential PAF, the same issues (in particular risk factors of interest acting as confounders of causally downstream risk factors of interest) mentioned in the section above can also cause biases in estimating sequential PAF and average PAF. These can again be handled by recursive application of the do-operator and simulation from the corresponding distributions. Practically sequential PAF may be of interest if population health interventions are to be applied incrementally (for instance, what would be the next risk factor to target in a health intervention after a successful intervention that targets smoking?), but another use is in the definition and estimation of average population attributable fractions, again first introduced in [23].

As explained above, individual population attributable fractions for differing risk factors in a set are not expected to sum to the joint PAF corresponding to eliminating all risk factors in the set. Over the years, differing proposals have been made to contstruct versions of attributable fractions for individual risk factors that do form a partition for the joint PAF (that is they sum up to the corresponding joint PAF). The most convincing of these constructions are average population attributable fractions [23]. Again suppose there are *K* risk factors, labeled again $\{1\ldots K\}$. Imagine eliminating these *K* risk factors in some sequence. This can be done in *K*! different ways. Each of these *K*! permutations can be represented as $\sigma = \sigma (1),\ldots ,\sigma (K)$, where $\sigma (j)=k$ if the risk factor *k* is the $j^{th}$ risk factor eliminated according to that ordering, and as such each permutation is associated with a sequential PAF for each risk factor. For instance, in the previous example the sequential PAF for risk factor *k* according to $\sigma$ would be $SAF_{k\mid \{\sigma (1)\ldots \sigma (j-1)\}}$ if $j \ge 2$ or just the *PAF* for risk factor *k* if $j=1$. The average PAF, $APAF_k$, for risk factor *k* is the average of the sequential *PAF*s over all *K*! different permutations. By definition, the sequential PAFs for differing risk factors corresponding to a particular permutation must add to the joint PAF. From this it follows easily that the average of these sequential PAFs for each risk factor across differing permutations (that is the average PAF) must also add over differing risk factors to the joint PAF.

### Estimation

At first look, it seems that one must calculate *K*! differing sequential PAFs to calculate average PAF for a risk factor. However, examining (24) we see that any sequential PAF is the difference between two differing joint PAFs. The number of joint PAF calculations is the same as the number of non-empty subsets of $\{1\ldots K\}$ (that is $2^K-1$, much smaller than *K*!). Provided the number of risk factors isn’t too large (say 10 or fewer) this it is quite feasible to calculate all possible sequential PAFs utilizing this approach. Average PAF for risk factor $k \le K$ can then be calculated using:

25 $$\begin{aligned}{} & {} APAF_{k}\nonumber \\{} & {} = \frac{\sum _{j=1}^{K}(K-j)!(j-1)!\sum _{{\textbf{S}} \subset \{1,\ldots ,K\}\setminus k: \mid {\textbf{S}}\mid =j-1}PAF_{k\mid {\textbf{S}}}}{K!}. \nonumber \\ \end{aligned}$$

The ‘exact’ approach to estimating $APAF_k$ is to first estimate $PAF_{k \mid {\textbf{S}}}$ for all possible subsets: ${\textbf{S}}\subset \{1,\ldots K\} \setminus k$ of risk factors sets that exclude *k*, and then plug these estimates into (25). This is done most efficiently when calculating *APAF* for all *K* risk factors together.

When $2^K$ is very large, estimating (25) exactly may be too time consuming. Recognizing instead that $APAF_k$ is a ‘population’ average of *K*! sequential PAFs, each sequential PAF corresponding to a single permutation (with admittedly many of these permutations lead to the same SAF), one can approximate the *APAF* by randomly sampling a smaller number nperm $< K!$ of permutations. Obviously, the larger nperm is, the smaller the approximation error from this step, which like any sample average decreases probabilistically at rate $\frac{1}{\sqrt{\text {nperm}}}$ as nperm increases. In practice, nperm$=1000$ has been suggested to achieve acceptable accuracy [8]. Stratified sampling of permutations (ensuring for instance that each risk factor appears in position 1 in the elimination order an equal number of times in the nperm permutations) can somewhat reduce the approximation error. We will describe this in the next section.

### Examples

Let’s extend the example from earlier where we looked at the joint PAF for smoking and high_blood_pressure, to include a 3rd risk factor diabetes. Note that lipids and waist_hip_ratio are joint causes of diabetes and stroke (see Fig. 5), and we now need to extend our causal graph and associated list of statistical models to include these variables in addition to diabetes. 
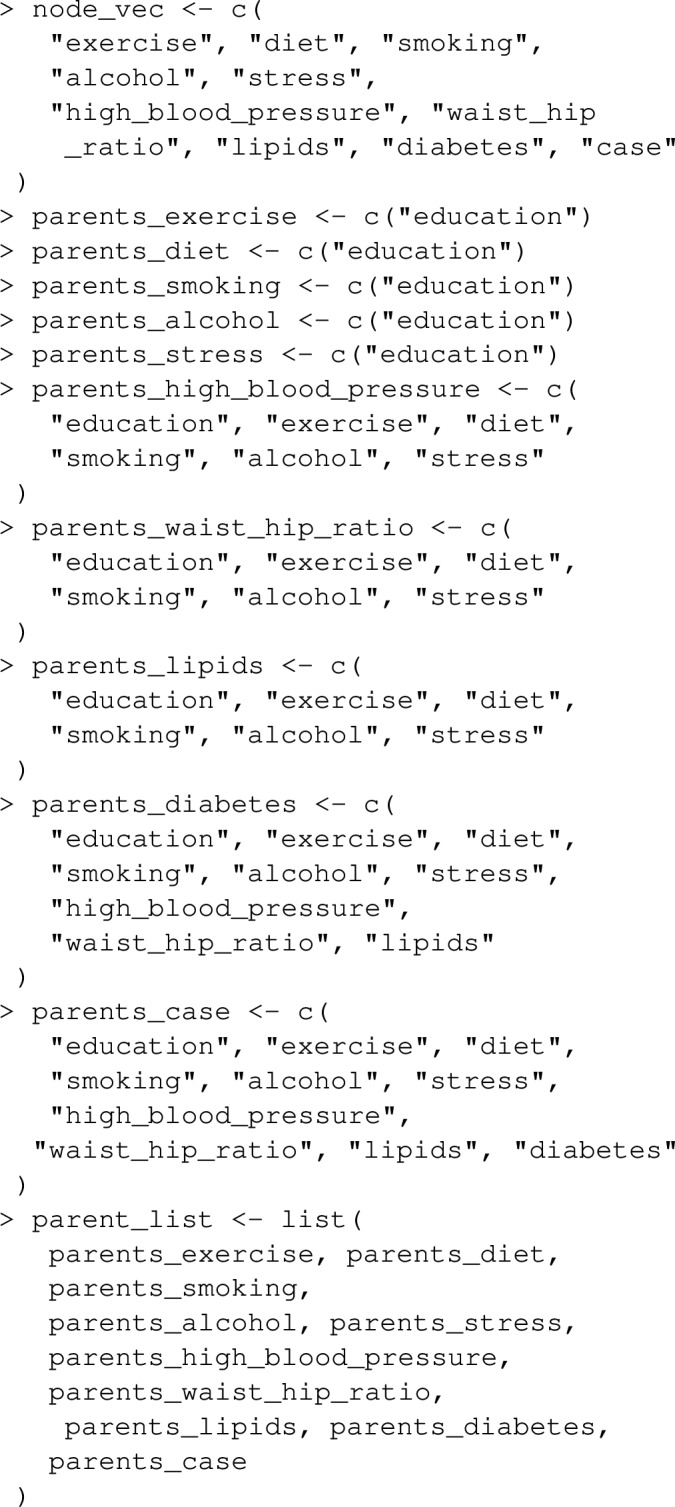
 Again we can automatically specify models using the automatic_fit function which now will fit models for the extra variables specified in node_vec. Note that lipids and waist_hip_ratio are also continuous risk factors and we can allow non-linear effects by adding these variable names to the spline_nodes argument. 
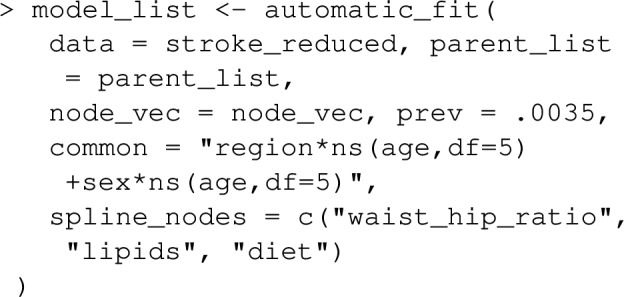
 Single sequential PAFs can be estimated with the function seq_paf, which has the same structure as joint_paf. The most important argument is vars, a vector of risk factors. Sequential PAF is estimated for the risk factor specified in the last position of vars conditional on the risk factors in earlier positions. For instance, the code below estimates sequential PAF for eliminating diabetes, in a population where smoking and high_blood_pressure are already eliminated. As can be seen below, this estimator is randomised: the estimate varies slightly based on the simulated data. The reason for this is that now the discrete variable diabetes is included in the dataset: ${\textbf{D}}_{\{\text {smoking, high\_blood\_pressure}\}}$, and the simulated value for diabetes under interventions for smoking and high_blood_pressure will vary slightly from simulation to simulation. Nevertheless as demonstrated below, the variation over simulation repetitions is fairly minimal and this variability will be accounted for in the Bootstrap confidence interval. Overall, this analysis suggests that in a population where smoking and high_blood pressure were already eliminated, an extra 2.3% of strokes (taken as a percentage of the number of strokes in the current population) might be prevented if there was no diabetes. 
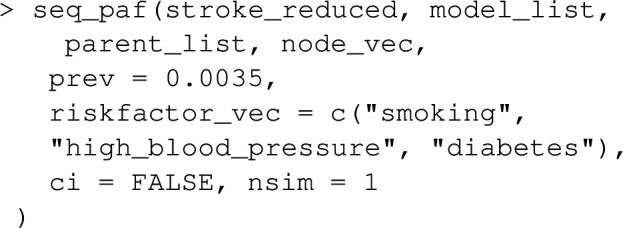

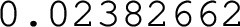

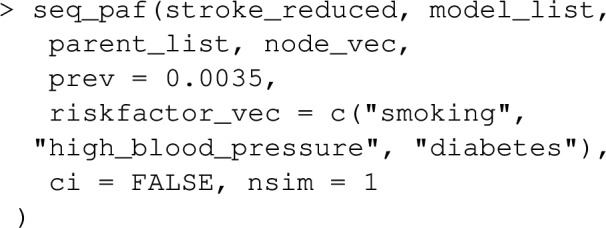

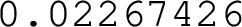

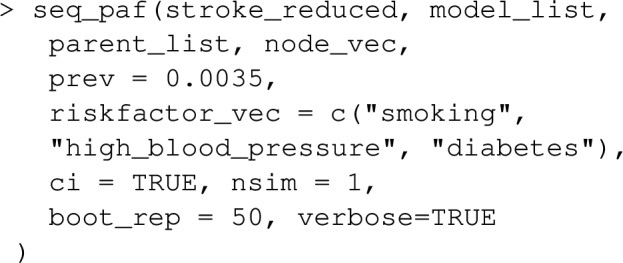

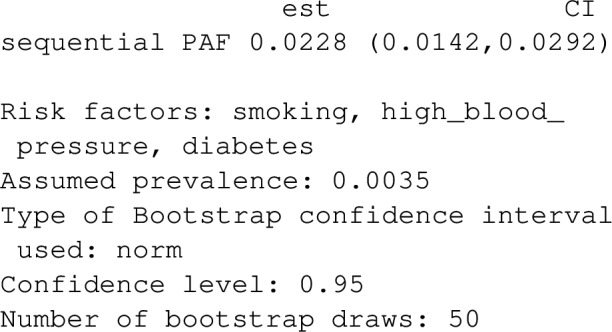
 The function average_paf generates results for average PAF for the three risk factors: smoking, high_blood _pressure and diabetes. The default estimation method is to first estimate joint PAF for all possible risk factor subsets, ${\textbf{S}}$, next to estimate all sequential PAFs, $PAF_{j\mid {\textbf{S}}}$, from the vector of joint PAFs and finally substitute these estimated sequential PAF into (25). Recall that in estimating joint PAF for the risk factor set ${\textbf{S}}$, a data set $D_{{\textbf{S}}}$ corresponding to this joint intervention is simulated recursively. The recursive nature of this simulation can be exploited to perform the estimation of all $2^K$ joint PAFs efficiently. For instance, when simulating data: $D_{{\textbf{S}} \cup \{j\}}$ corresponding to eliminating risk factors: ${\textbf{S}} \cup \{j\}$, with *j* being the final risk factor eliminated, data corresponding to eliminating the risk factors in ${\textbf{S}}$, $D_{{\textbf{S}}}$ has already been simulated. average_paf calculates joint PAF for the $2^K$ risk factor subsets in an order that allows extensive use of this fact. As illustrated in the results below, estimated average PAF is highest for high_blood_pressure at 0.264, with smoking at 0.101 and diabetes at 0.0391. In addition, average sequential PAF by elimination position for each risk factor is provided. Note that the sequential PAF for diabetes is most effected by elimination position. This makes sense based on its position in the causal graph (causally upstream of smoking and high_blood_pressure) 
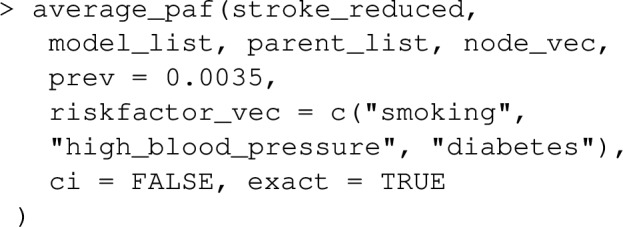

**Fig. 7:**
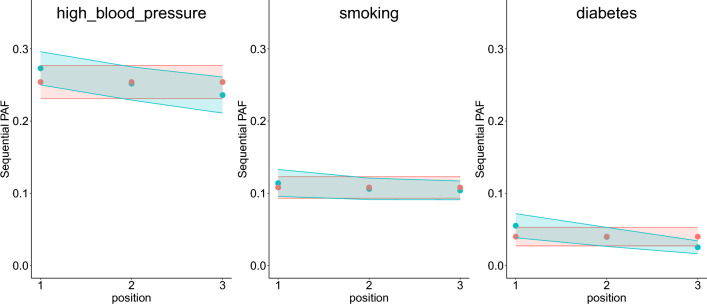
Estimated average PAF and sequential PAF for the group of risk factors: smoking, blood pressure and diabetes. Risk factors are plotted in decreasing order of estimated *APAF*. Average *PAF* is shaded in pink, and the average sequential PAF for particular risk factors by elimination position in blue. One expects sequential PAF to decrease over elimination position which is what we observe here


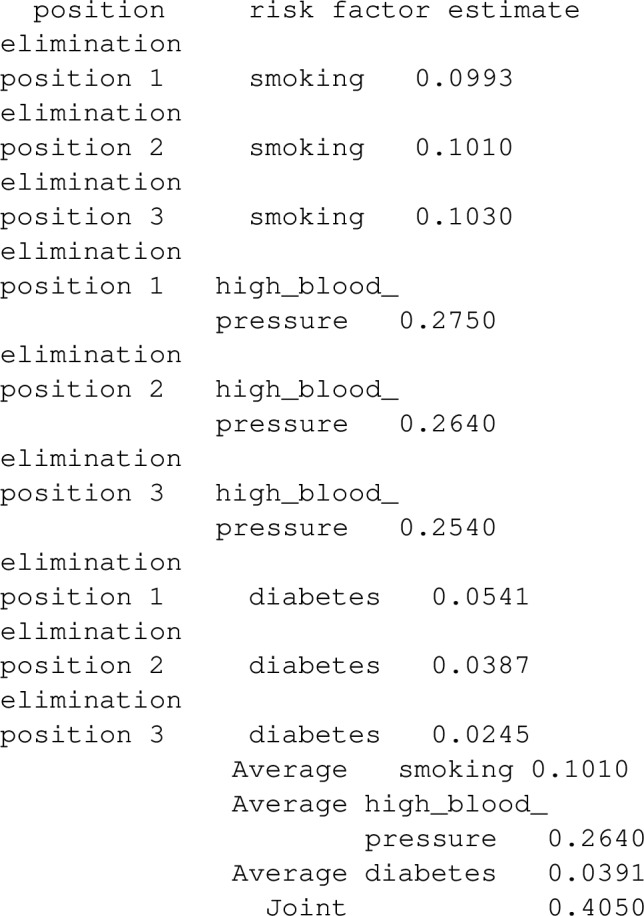
 In the above analysis, the estimator is again randomised. While all $2^K-1$ joint PAFs need to be estimated to enable this calculation for all risk factors; each estimated joint PAF corresponds to a single simulated data set $D_{{\textbf{S}}}$, which can generate substantial Monte Carlo variability for small datasets and small *K*. As an alternative, one can sample nperm differing permutations of $\{1,\ldots ,K\}$: corresponding to differing risk factor elimination orders, calculate sequential PAFs associated with each permutation and average the associated sequential PAF for a particular risk factor. For small *K* and nperm $>2^K$ this approach is likely to have reduced Monte Carlo error (compared to this default). If the argument exact=FALSE, this approach is used in place of the estimator based on (25), with the number of randomly sampled permutations controlled by the argument nperm. Stratified sampling of permutations (so that the joint empirical distribution of permutation positions $\sigma (1),\ldots ,\sigma (S)$ for some $S < K$ is uniform (as it would be if we calculated sequential PAFs for all *K*! permutations), can help further reduce Monte Carlo error. For *K* risk factors, an integer multiple of $K(K-1)\ldots (K-S+1)$ permutations are needed to implement such a strategy. Such stratified sampling of permutations is implemented through the argument correct_order (for instance, correct_order=*S* in the preceding example).

In contrast, for a large number of risk factors, *K*, averaging sequential PAF over a number of randomly sampled permutations nperm$< 2^K$ may be less accurate than estimating (25) directly, due to the Monte Carlo error associated with sampling permutations. However, it may be the only computationally viable option.

When confidence intervals are not requested an upper bound on the margin of error of the point estimate (in terms of how close to the calculation with nperm = $\infty$) is given (with 95%) confidence as calculated in [8], provided permutations are sampled (that is, when using the argument exact=FALSE). Note that this margin of error assumes non-stratified sampling rather than the more accurate stratified sampling implemented here. The results below indicate that the three average PAFs are calculated to within an accuracy of 0.004 (with 95% confidence) compared to the exact estimate when nperm $\rightarrow \infty$. 
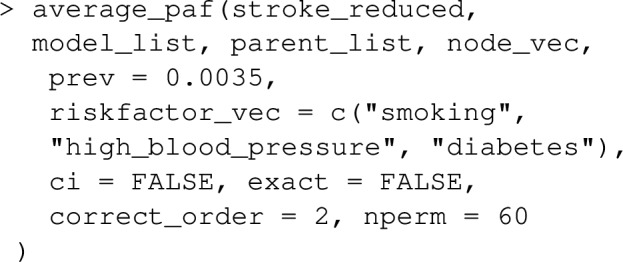

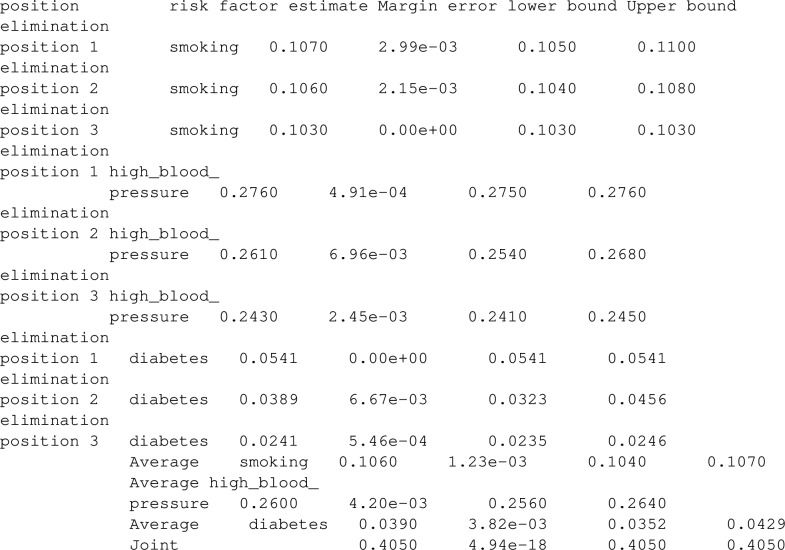


Of courses, sampling error also needs to be accounted for when making a statement about estimation accuracy. While in this case with only $K=3$ risk factors, estimation with 60 permutations should give a slightly more accurate point estimate for average PAF compared to estimation using equation (25) directly, the approximation error in both cases is much smaller than the sampling error. In fact, confidence intervals suggest comparable accuracy of using equation (25) (full_results_a) and calculating average PAF using 60 sampled permutations with stratified sampling (full_results_b) (Fig. 7).
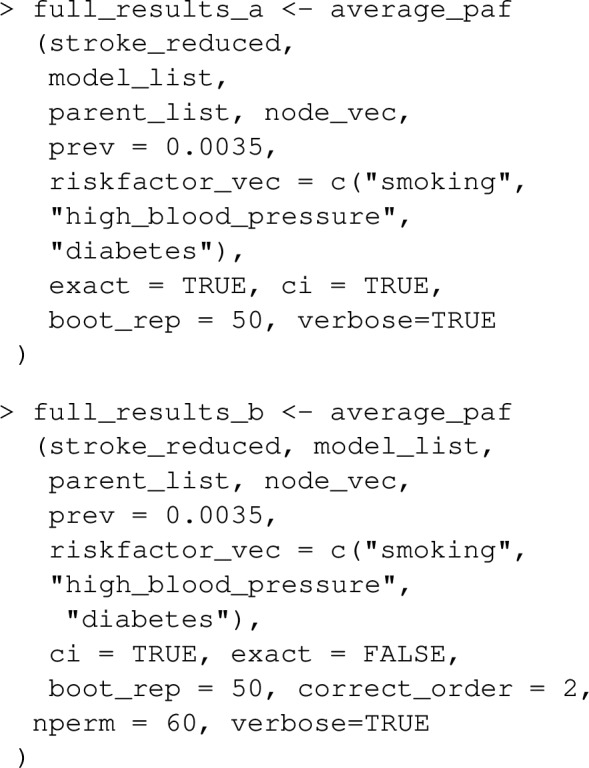

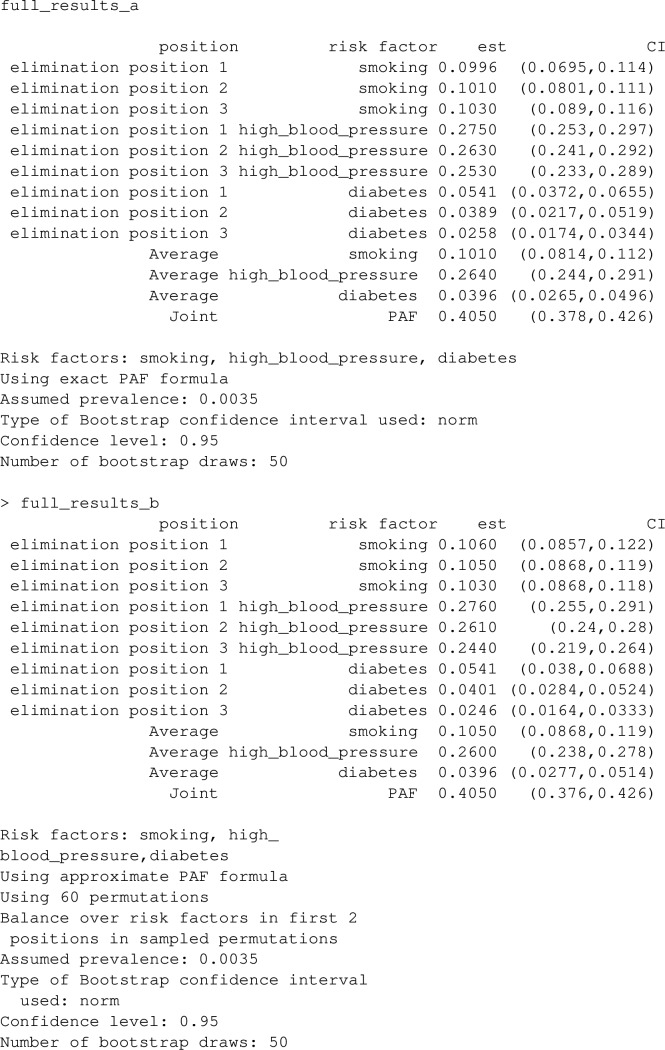


Results (average PAF and sequential PAF by elimination position, along with associated variability bands) can be plotted over differing risk factors as follows (see Fig. 7): 
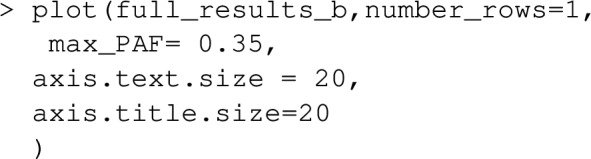


Note that if exact=FALSE and ci=FALSE, the plotted variability bands will not be interpretable as confidence intervals, but rather as bands for the degree of possibility approximation error in the point estimate.

### Computational considerations

As described here, graphPAF, facilitates incorporation of causal structure into estimation of joint, sequential and average PAF, essentially by incorporating recursive simulation methods based on an assumed causal structure. Ignoring such causal structure, as other approaches have in the past (for example, [8, 29]) may lead to bias. A drawback of this simulation based strategy is computational cost. Techniques such as Bootstrap-parallelization (through the boot package), intelligent ordering of calculations when calculating joint PAF for differing risk factor subsets, stratified sampling of permutations when the number of risk factors is large and the use of the more efficient formula for average PAF (25) can somewhat reduce these computational requirements. Computational cost depends jointly the size of the Bayesian networks and the size of the underlying dataset. The dataset stroke_reduced used in this manuscript has 13,712 rows and the algorithms described here can be run in reasonable time on most modern laptops when using this data. For larger datasets, splitting into independent subsets and rerunning these methods independently on each subset before averaging might be recommended to avoid memory management problems.

## Conclusions

In addition to implementing standard PAF estimation, graphPAF collates many recently developed tools for estimation of disease burden in non-standard settings into one package. We hope it will be useful to statisticians and epidemiologists who are interested in comparisons of disease burden over multiple risk factors, both discrete and continuous.

## Supplementary material

The most up to date version of graphPAF can be downloaded from the corresponding author’s Github repository: www.github.com/johnfergusonNUIG/graphPAF. The graphPAF package is also available to download from CRAN at https://CRAN.R-project.org/package=graphPAF
